# Supplementary material for: RNA-Seq Transcriptome Analysis and Evolution of OsEBS, a Gene Involved in Enhanced Spikelet Number per Panicle in Rice
Source: Int J Mol Sci. 2023 Jun 18;24(12):10303. doi: 10.3390/ijms241210303 (PMC10299296; doi:10.3390/ijms241210303)
Supplement: Supplementary file 1 [file ijms-24-10303-s001.zip › Figure S1-S2 and S5-S6.pdf]

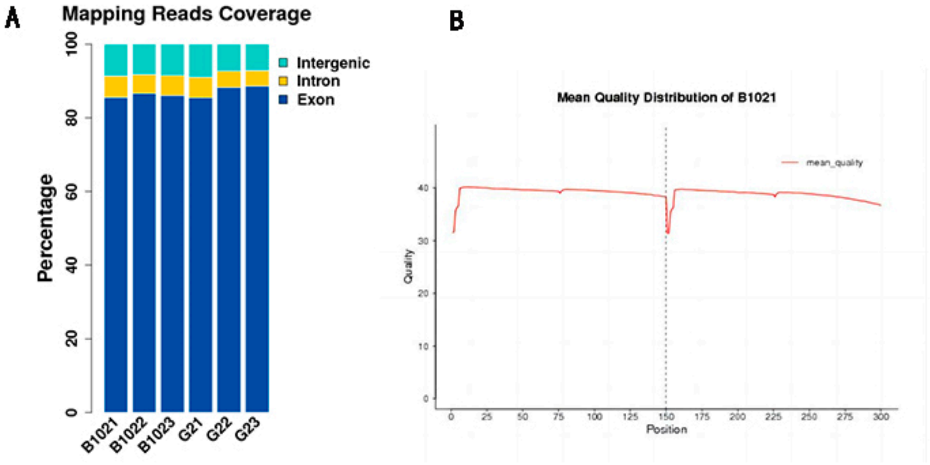

Figure S1. Quality control results after sequencing data alignment. **(A)** Distribution of the unique alignment sequence in each region of the reference genome gene. **(B)** uniformity analysis chart of B1021.

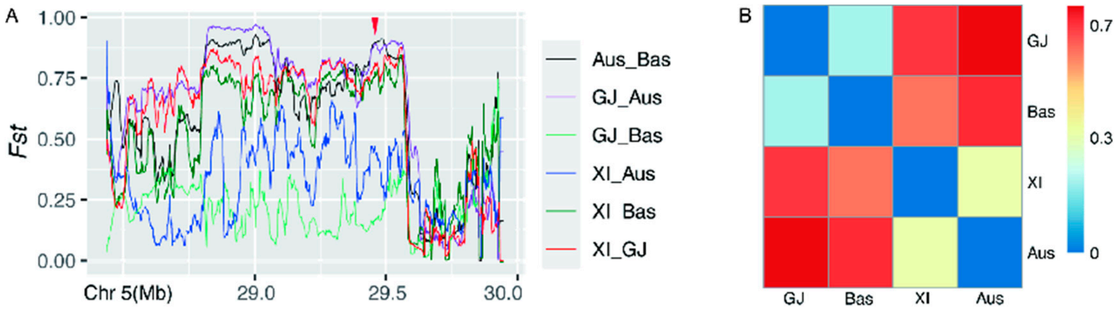

Figure S2. The degree of differentiation between different subspecies based on Fst analysis and the heat map. **(A)** The degree of differentiation between different subspecies based on Fst analysis. **(B)** The degree of differentiation between different subspecies based on the heat map.

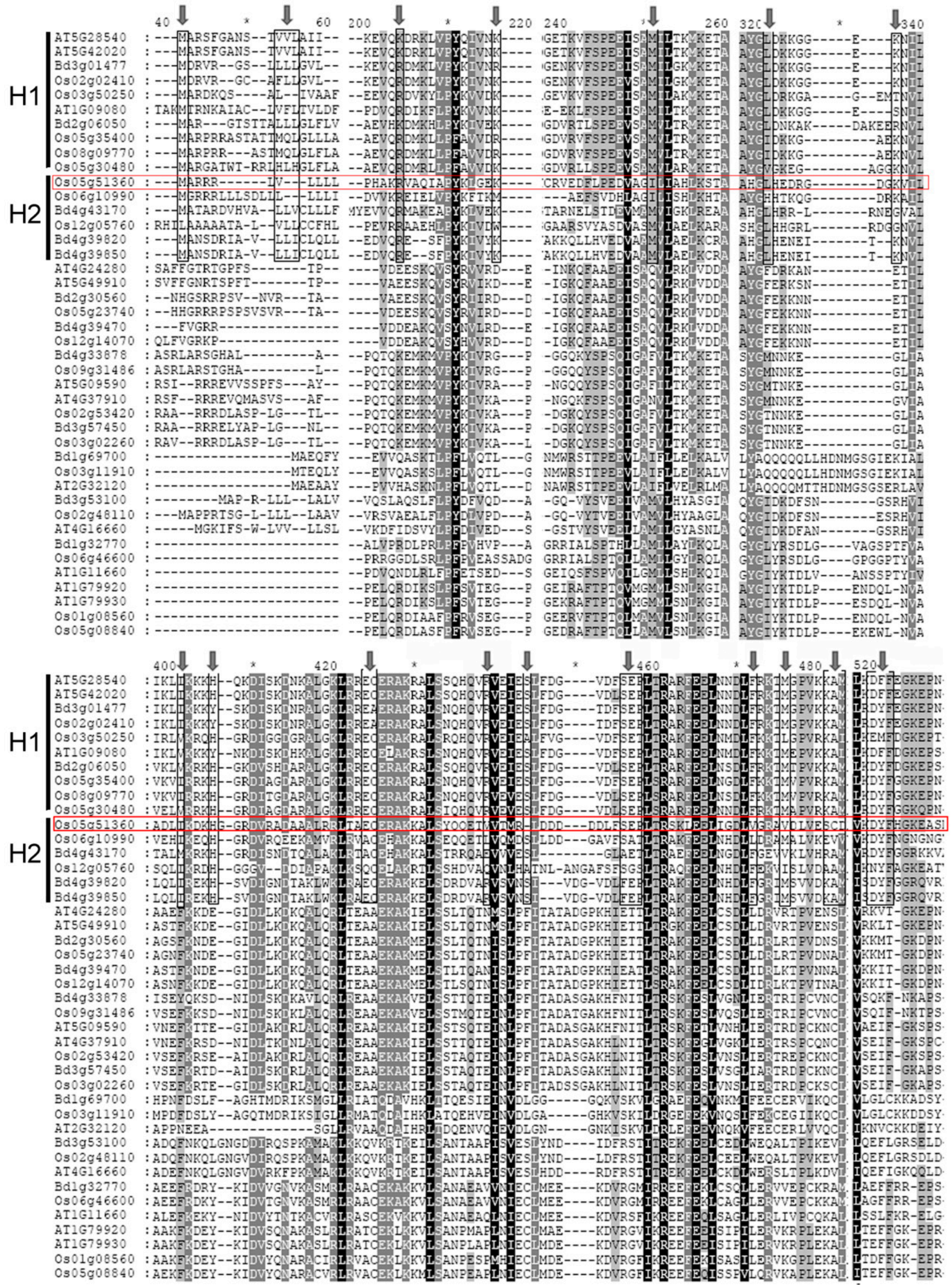

Figure S5. Multiple sequence alignment of OsEBS and other known Hsp70 proteins from *Oryza sativa* (Os), *Brachypodium distachyon* (Bd) and *Arabidopsis thaliana* (AT).

Red squares show the OsEBS (Os05g51360) sequence, black square brackets and gray arrow show consensus residues among H1 and H2 subfamily only. Black shading indicates consensus residues common to 42 Hsp70 proteins. Gray shading indicates consensus residues across 26 to 41 protein sequences. Accession numbers for 42 aligned sequences are shown on left. Numbers above figure indicate amino acid position. Sequences were aligned using ClustalW software and displayed using MEGA5.

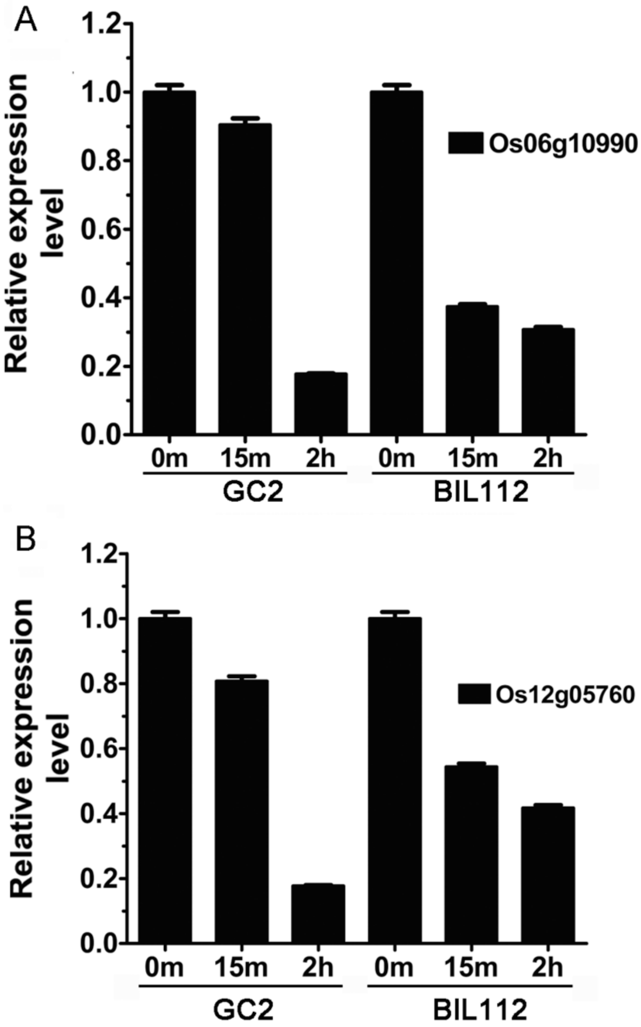

Figure S6. Expression pattern of Os06g10990 and Os12g05760 under heat shock treatment.

(**A**) qRT-PCR analysis of Os06g10990 expression under heat shock treatment. Seedlings (15-day old) of Guichao2 and BIL112 plants were subjected to heat shock treatment and samples were collected for analysis after 0 and 15 min, and 2 h. Values are means ± SD; n = 3 individuals and show expression level relative to that of *OsActin.*

(**B**) qRT-PCR analysis of Os12g05760 under heat shock treatment. Seedlings (15-day old) of Guichao2 and BIL112 plants were subjected to heat shock treatment and samples were collected for analysis at 0 and 15 min, and 2 h. Values are means ± SD; n = 3 individuals and show expression level relative to that of Os*Actin.*
