# Supplementary material for: RNA-Seq Transcriptome Analysis and Evolution of OsEBS, a Gene Involved in Enhanced Spikelet Number per Panicle in Rice
Source: Int J Mol Sci. 2023 Jun 18;24(12):10303. doi: 10.3390/ijms241210303 (PMC10299296; doi:10.3390/ijms241210303)
Supplement: Supplementary file 1 [file ijms-24-10303-s001.zip › Figure S3.pdf]

**Figure S3.** Clustal X (1.83) multiple sequence alignment.

## CLUSTAL X (1.83) MULTIPLE SEQUENCE ALIGNMENT

**File: Z:Hsp reeA1.pep.ps** **Date: Tue Dec 18 15:07:21 2012**



```
                                                                                                            . .. .*.     .                         : .          . .
AT5G28540   ----------MARSFGANS---------------TVVLAII-FF------GCLFAL------------------------SSAIEEATKLGSVIGIDLGTTYSCVGVYK----NGHVEII-ANDQGNRITPSWVGFTD---SERLIGEAA   86
AT5G42020   ----------MARSFGANS---------------TVVLAII-FF------GCLFAF------------------------STAKEEATKLGSVIGIDLGTTYSCVGVYK----NGHVEII-ANDQGNRITPSWVGFTD---SERLIGEAA   86
Bd3g01480   ----------MDRV--RGS---------------LLLLGVL-LA------GSLFAF------------------------SAAKEEAKKLGTVIGIDLGTTYSCVGVYK----NGHVEII-ANDQGNRITPSWVGFTD---SERLIGEAA   84
Os02g02410  ----------MDRV--RGC---------------AFLLGVL-LA------GSLFAF------------------------SVAKEETKKLGTVIGIDLGTTYSCVGVYK----NGHVEII-ANDQGNRITPSWVAFTD---SERLIGEAA   84
AT1G09080   MIFIKENTAKMTRNKAIAC---------------LVFLTVL-DFLM-NIGAALMSS------------------------LAIEGEEQKLGTVIGIDLGTTYSCVGVYH----NKHVEII-ANDQGNRITPSWVAFTD---TERLIGEAA  101
Os03g50250  ----------MARDKQSAL-----------------IVA---AFVL-LCSGCLCGV-----------------------ADGAKGGRKTKGPVIGIDLGTTYSCVGVYR----NGHVDIV-ANDQGNRITPSWVAFTD---DERLVGEAA   88
Bd2g06050   ----------MARGTST----------------TALLLGLF-LVGFLVAPSAAAADS-----------K----------GDQQPANKASGGPVIGIDLGTTYSCVGVYR----NGHVEII-ANDQGNRITPSWVAFTD-S-GERLIGEAA   95
Os05g35400  ----------MARPRRASTA-------------TTMQLGLL-LAALLLFTSSLAGSV-----------AAAAPPPPAGAKGGGAKSGGGGGTVIGIDLGTTYSCVGVYR----NDRVEII-ANDQGNRITPSWVAFTD-G-GERLIGEAA  108
Os08g09770  ----------MARPRRASTM----------------QLGLF-LAALLLLTPSPAGSV-----------AA---------AKGGGAKSGGGGTVIGIDLGTTYSCVGVYR----NGHVEII-ANDQGNRITPSWVAFTD-G-GERLIGEAA   96
Os05g30480  ----------MARGATWTR--------------RLHLHGLF-LAVLLLLTLP---------------------------AGSTAAAGGGGGTVIGIDLGTTYSCVGVYR----NGHVEII-ANDQGNRITPSWVAFTG-G-GERLIGEAA   91
Os05g51360  ----------MARRRLVLL-------------------------LLII----------AAAGAAAFGIRRDAPKAYCFYGIEWTGVPYLPGTAAAIYIGNTNSCIAGYH----SPPD----TATSYRFCIPSWVAFTA-N-ATTLCGQPA   95
Os06g10990  ----------MGRRRLLLS-------------------DLLLLLLIAIAVAGAAAAIAIPRAAPAFGVETGWPPEHCLRCFAPPDAPFVLGAAAAIHLGNTNSCIAGYD----DDDAPLGAKRSYYQFCIPSWVALAHDN-GTVISGEAA  116
Bd4g43170   ----------MATARDVHV---------------ALLVCLL-------------LFV-----------FSGQPQPASASKSGKRCYRFRSPPVVALDIGNTNSCIAGYV----ADG-----SDAMFQLCIPSWVAFTA-N-GTILVGDDA   90
Os12g05760  -----MARHILAAAA-ATA---------------LVLLCCF-------------HLP-----------ATAM--MADTIRYGPPRVPHRLGTVIAVDLGNTNSCVAGYG----DEAD----APPLFRLCIPTSVAFTG-D-GDALVGEAA   93
Bd4g39820   ----------MANSD-RIA---------------VLLICLQ-------------LLP-----------SGFLGHAAALMDSPLPSRPFRLGSVIAVRLGNTRSCVSGYG----NGHA----GAPVFRFCIPSWVAFSD-D-GAALVGEAA   90
Bd4g39850   ----------MANSD-RIA---------------VLLICLQ-------------LLP-----------SGFLGHAAALMDSPLPSRPFRLGSVIAVRLGNTRSCVSGYG----NGHA----GAPVFRFCIPSWVAFSD-D-GAALVGEAA   90
AT4G24280   MASSAAQIHVLGGIGFASSSS--SKRNLNGKGGTFMPRSAFFGTRTGPFSTPTSAFL-----------RMGTRNGGGASRYAVGPVRVVNEKVVGIDLGTTNSAVAAME----GGKPTIV-TNAEGQRTTPSVVAYTK-S-GDRLVGQIA  130
AT5G49910   MASSAAQIHILGGIGFPTSSSSSSTKNLDNKTNSI-PRSVFFGNRTSPFTTPTSAFL-----------RMGRRNN-NASRYTVGPVRVVNEKVVGIDLGTTNSAVAAME----GGKPTIV-TNAEGQRTTPSVVAYTK-S-KDRLVGQIA  130
Bd2g30560   ----------MATTTFPTSTPFFVNH----------------GSRRPSVNV----RT-----------AA--AVY-GRGGRRWRPLRVACEKVVGIDLGTTNSAVAAME----GGKPTIV-TNAEGARTTPSVVAYTK-A-GDRLVGQIA   99
Os05g23740  ----------MATTTFPTSTPFFAHH----------------GRRRPSPSV----SV-----------RTAAAVYGRGGGRRWRPLRVACEKVVGIDLGTTNSAVAAME----GGKPTIV-TNAEGARTTPSVVAYTK-S-GDRLVGQIA  102
Bd4g39470   MATFTPQVSAMACGSPSSS--------------------LFVGRRRRP-ALQM--------------------RAPRGGRARGLAMRVACEKVVGIDLGTTNSAVAAME----AGKPTVI-TNAEGQRTTPSVVAYTK-G-GERLVGQIA  102
Os12g14070  MASFTSQLGAMACGAAPSTSPLAARRSG----------QLFVGRKPAAASVQM--------------------RVPRAGRARGVAMRVACEKVVGIDLGTTNSAVAAME----GGKPTVI-TNAEGQRTTPSVVAYTK-G-GERLVGQIA  113
Bd4g33880   MAIGSLVASRLARSGH-------------------------ALATAAISQAPRSRHT------------TSPLLSRLGAVARAFSSKPAAADVIGIDLGTTNSCVSVMD----GKTPRVI-ENAEGARTTPSIVAKNQ-N-GDLLIGITA  106
Os09g31486  MAIGSLIASRLARSTG-------------------------HALASAASQAPMARHA-----------AASPLLSRLGSVARAFSSKPAAADVIGIDLGTTNSCVSVME----GKTPRVI-ENAEGARTTPSIVAKNQ-N-GDLLVGITA  107
AT5G09590   MATAALLRSIRRREVVS-------------------S--PFSAYRCL--SSSGKASL-----------NSSYLGQNFRSFSRAFSSKPAGNDVIGIDLGTTNSCVAVME----GKNPKVI-ENAEGARTTPSVVAFNT-K-GELLVGTPA  109
AT4G37910   MASVALLRSFRRREVQM-------------------A--SVSAFKSV--SANGKNSM-----------F-----GKLGYLARPFCSRPVGNDVIGIDLGTTNSCVSVME----GKTARVI-ENAEGSRTTPSVVAMNQ-K-GELLVGTPA  104
Os02g53420  MAASLLLRAARRRDLAS-------------------P--LGTLTANA--QSA----------------YSANICSQWGSFARAFSVKPTGNEVIGIDLGTTNSCVSVME----GKNPKVI-ENSEGTRTTPSVVAFNQ-K-GERLVGTPA  104
Os03g02260  MAASLLLRAVRRRDLAS-------------------P--LGTLTANV--QSK----------------CAANVCSRWAGFARTFSAKATGNEVIGIDLGTTNSCVSVME----GKNPKVI-ENSEGTRTTPSVVAFNQ-K-GELLVGTPA  104
Bd3g57450   MAASLLLRAARRRELYA-------------------P--LGNLTTNV--QST----------------FATNACSRWGGFARAFSAKPIGNEVIGIDLGTTNSCVSVME----GKNAKVI-ENSEGTRTTPSVVAFSQ-K-GERLVGTPA  104
Bd1g69700   -------------------------------------MA-----------------------EQF---YTVASDSETTGEE--KAQQTFPDVAIGIDIGTSKCSVAVWN----GHQVELL-KNTRSQKGMRSYVMFKDDTLSAGVTGGAT   80
Os03g11910  -------------------------------------MT-----------------------EQL---YTVASDSETTGED--KSQMSFPDVAIGIDIGTSNCSVAVWT----GHQVELL-KNTRNQKGMRSYVMFKDDSLSAGVTGGAT   80
AT2G32120   -------------------------------------MA-----------------------EAA---YTVASDSENTGEEKSSSSPSLPEIALGIDIGTSQCSIAVWN----GSQVHIL-RNTRNQKLIKSFVTFKDEVPAGGVSNQLA   82
Bd3g53100   ----------MAPR-----------------------------LLL------------------------------LALVVAAVAVPPASAAVASIDLGSEWLKVAAVHLAPGRAPIAVA-INEMSKRKSPALAALAD---GNRLAGEEA   77
Os02g48110  ----------MAPPRTSGL----------------LL-----LLAA------------------------------VVAAAAVVLVPPAEAAVASIDLGSEWLKVAAVHLAPGRVPIAVA-INEMSKRKSPALAALAD---GNRLAGEEA   85
AT4G16660   ----------MGKIFS---------------------------WLV------------------------------VLLSLISLVPVPSESAVLSVDLGSEWVKVAVVNLKRGQSPISVA-INEMSKRKSPALVAFQS---GDRLLGEEA   79
Bd1g32770   ------------------------------------------------------------------------------------------MSVVGFDVGNDTLVAAAAR----QRGIDVL-LNAESKRESPAAVAFAH---NARLLGPHA   52
Os06g46600  ------------------------------------------------------------------------------------------MSVVGFDVGNDTLVAAAAR----QRGIDVL-LNAESNRESPAAVAFSH---NARLLGPHA   52
AT1G11660   ------------------------------------------------------------------------------------------MSVVGFDVGNENCVIAVAK----QRGIDVL-LNDESNRENPAMVSFGE---KQRFMGAAA   52
AT1G79920   ------------------------------------------------------------------------------------------MSVVGFDFGNENCLVAVAR----QRGIDVV-LNDESNRETPAIVCFGD---KQRFIGTAG   52
AT1G79930   ------------------------------------------------------------------------------------------MSVVGFDFGNENCLVAVAR----QRGIDVV-LNDESNRETPAIVCFGD---KQRFIGTAG   52
Os01g08560  ------------------------------------------------------------------------------------------MSVVGFDLGNESCIVAVAR----QRGIDVV-LNEESKRETPAIVCFGD---KQRFIGTAG   52
Os05g08840  ------------------------------------------------------------------------------------------MSVVGFDVGNESGIVAVAR----QRGIDVV-LNEESKRETPAVVCFGD---KQRFIGTAG   52
ruler       1.......10........20........30........40........50........60........70........80........90.......100.......110.......120.......130.......140.......150
```

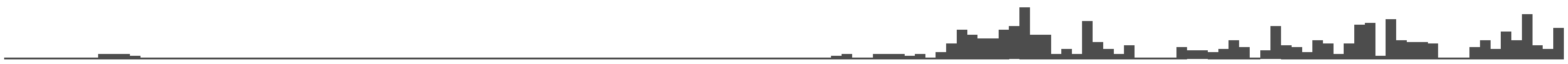

# CLUSTAL X (1.83) MULTIPLE SEQUENCE ALIGNMENT

**File: Z:Hsp reeA1.pep.ps** **Date: Tue Dec 18 15:07:21 2012**



```
                                 :                   .:                                   .: . .:      ..         :    . :*                 :   .*.    :. :  . :
 AT5G28540 KNQAA----VN-PERTVFDVKRLIGRKFED--KEVQKDRKLVPYQIVN---K-DG-KPYIQVKI----KDGETKVFSPEEISAMILTKMKETAEAYL---GKKIKDAVVTVPA---YFNDAQRQATK-DAGVIAGLNVARIINEPTAAAI  213
 AT5G42020 KNQAA----VN-PERTVFDVKRLIGRKFED--KEVQKDRKLVPYQIVN---K-DG-KPYIQVKI----KDGETKVFSPEEISAMILTKMKETAEAYL---GKKIKDAVVTVPA---YFNDAQRQATK-DAGVIAGLNVARIINEPTAAAI  213
 Bd3g01480 KNQAA----VN-PERTIFDVKRLIGRKFED--KEVQRDMKLVPYKIVN---R-DG-KPYIQVKI----KDGENKVFSPEEISAMILGKMKETAEAYL---GKKINDAVVTVPA---YFNDAQRQATK-DAGVIAGLNVARIINEPTAAAI  211
 Os02g02410 KNQAA----VN-PERTIFDVKRLIGRKFED--KEVQRDMKLVPYKIVN---K-DG-KPYIQVKI----KDGENKVFSPEEVSAMILGKMKETAEAYL---GKKINDAVVTVPA---YFNDAQRQATK-DAGVIAGLNVARIINEPTAAAI  211
 AT1G09080 KNQAA----KN-PERTIFDPKRLIGRKFDD--PDVQRDIKFLPYKVVN---K-DG-KPYIQVKV----K-GEEKLFSPEEISAMILTKMKETAEAFL---GKKIKDAVITVPA---YFNDAQRQATK-DAGAIAGLNVVRIINEPTGAAI  227
 Os03g50250 KNQAA----LN-PDRTIFDIKRLIGRRFDD--EEVQRDVKYLPYKVVD---K-GG-KPYVEVRV----KAGEVKVFSPEEISAMILAKMKETAESYL---GQRVTDAVVTVPA---YFNDAQRQATK-DAGTIAGLNVPRIINEPTAAAI  215
 Bd2g06050 KNQAA----SN-PLRTIYDAKRLIGRQYGD--AEVHKDMKHLPYKIVE---K-RG-KPHMEVEV----KDGDVRTLSPEEVSAMVLTRMKETAEAFL---GEPVKDAVITIPA---YFNDAQRQATK-DAGAIAGLNVVRLINEPTAAAI  222
 Os05g35400 KNQAA----AN-PERTIYDAKRLIGRQFSD--AEVQRDMKLLPFAVVD---R-NG-KPHVRVEV----KDGDVRVFSPEEVSAMVLTRMKETAEAYL---GEKVTRAVVTVPA---YFNDAQRQATK-DAGVIAGLTVDRIINEPTAAAI  235
 Os08g09770 KNQAA----AN-PERTIYDAKRLIGRQFSD--AEVQRDMKLLPFAVVD---R-NG-KPHVRVEV----KDGDVRVFSPEEVSAMVLTRMKETAEAYL---GEKVTRAVVTVPA---YFNDAQRQATK-DAGVIAGLTVDRIINEPTAAAI  223
 Os05g30480 KNQAA----AN-PGRTVYDAKRLIGRRFAD--AEVQRDMRLLPFAVVD---K-GG-KPHVRVEV----RGGDVRLLSPEEVSAMVLARMKETAEAYL---GEEVTRAVVTVPA---YFNDAQRQATK-DAATIAGLAVERILNEPTAAAL  218
 Os05g51360 VDH----------SAAISGFKRLIGLQPGD--PHAKRVAQIAPYKLGE---K-IG-RCSIQVQLDD-GAKCRVEDFLPEDVAGILIAHLKSTAEAHL---GHRIDNAVVTVPG---HFNGNQRQEVSSGSTEYGGFRYVSVVDEQVAAAA  221
 Os06g10990 MNRAA----LS-PSTAVSAFMRLLHRRVED--DVVKREIELVPYKFTK---M-LG-WVSVQLDTD-------AEFSVDHLAGILISHLKHTAEAHL---GRHINNAVITLPSRLSYSADG-RQVLSSAAKEYSGFRAVKVVDEHIAAAA  242
 Bd4g43170 QDYAA----VD-PASAVSGFKRLIGMRWSHMYEVVQRMAKEAPYKLVE---K-NL-YPHIKMKTGYIVGGTARNELSTDEVMAMVIGKLREAAESYM---SCSVRHAVFTVPR---HYYDSPWRQTE-FAGHIAGVRVARMLDEPIAAAV  223
 Os12g05760 KNHP----------AAISGFKRLLGTRFGS--PEVRRAAEHLPYKIVD---W-CT-MAHIEVNA---GAGGAARSVYASDVASMVIAELKARAEARLAGGGKKVHNAVVTVPY---YFSDGPREAAM-NAARMAGLTTVRIIDEPTAAAV  219
 Bd4g39820 KDHAA----AN-PQAAVFGFKRLLGKTFGH--EDVQRE--SFPYKIVY---K-ET-SSTGT--G---IQVAKKQLLHVEDVAAMVLAELKCRAEAFL---GHKVHYAVLTVPS---YFRDASRLAAF-DAVTSAGLEPVRIIDEPTAAAL  214
 Bd4g39850 KDHAA----AN-PQAAVFGFKRLLGKTFGH--EDVQRE--SFPYKIVY---K-ET-SSTGT--G---IQVAKKQLLHVEDVAAMVLAELKCRAEAFL---GHKVHYAVLTVPS---YFRDASRLAAF-DAVTSAGLEPVRIIDEPTAAAL  214
 AT4G24280 KRQAV----VN-PENTFFSVKRFIGRKMNE----VDEESKQVSYRVVR---DENN-NVKLECP-------AINKQFAAEEISAQVLRKLVDDASRFL---NDKVTKAVITVPA---YFNDSQRTATK-DAGRIAGLEVLRIINEPTAASL  253
 AT5G49910 KRQAV----VN-PENTFFSVKRFIGRRMNE----VAEESKQVSYRVIK---DENG-NVKLDCP-------AIGKQFAAEEISAQVLRKLVDDASRFL---NDKVTKAVITVPA---YFNDSQRTATK-DAGRIAGLEVLRIINEPTAASL  253
 Bd2g30560 KRQAV----VN-PENTFFSVKRFIGRKMNE----VAEESKQVSYRIIR---DDNG-NVKLDCP-------AIGKQFAAEEISAQVLRKLVDDASKFL---NDKVTKAVITVPA---YFNDSQRTATK-DAGRIAGLDVLRIINEPTAASL  222
 Os05g23740 KRQAV----VN-PENTFFSVKRFIGRKMNE----VDEESKQVSYRVIR---DDNG-NVKLDCP-------AIGKQFAAEEISAQVLRKLVDDASKFL---NDKVTKAVITVPA---YFNDSQRTATK-DAGRIAGLEVLRIINEPTAASL  225
 Bd4g39470 KRQAV----VN-PENTFFSVKRFIGRKMAE----VDDEAKQVSYNVLR---DENG-NVKLDCP-------AIGKQFAAEEISAQVLRKLVDDASKFL---NEKITKAVVTVPA---YFNDSQRTATK-DAGRIAGLEVLRIINEPTAASL  225
 Os12g14070 KRQAV----VN-PENTFFSVKRFIGRKMAE----VDDEAKQVSYHVVR---DDNG-NVKLDCP-------AIGKQFAAEEISAQVLRKLVDDASKFL---NDKITKAVVTVPA---YFNDSQRTATK-DAGRIAGLEVLRIINEPTAASL  236
 Bd4g33880 SRQAV----TN-AQNTVRGSKRLIGRTFDD--PQTQKEMKMVPYKIVR---GPNG-DAWVEM---------GGQKYSPSQIGAFVLTKMKETAESFL---GKTVSKAVITVPA---YFNDAQRQATK-DAGRIAGLEVMRIINEPTAAAL  229
 Os09g31486 SRQAV----TN-AQNTVRGSKRLIGRTFDD--PQTQKEMKMVPYKIVR---GPNG-DAWVEM---------GGQQYSPSQIGAFVLTKMKETAEAFL---GKTVSKAVITVPA---YFNDAQRQATK-DAGRIAGLEVMRIINEPTAAAL  230
 AT5G09590 KRQAV----TN-PTNTVSGTKRLIGRKFDD--PQTQKEMKMVPYKIVR---APNG-DAWVEA---------NGQQYSPSQIGAFILTKMKETAEAYL---GKSVTKAVVTVPA---YFNDAQRQATK-DAGRIAGLDVERIINEPTAAAL  232
 AT4G37910 KRQAV----TN-PTNTIFGSKRLIGRRFDD--PQTQKEMKMVPYKIVK---APNG-DAWVEA---------NGQKFSPSQIGANVLTKMKETAEAYL---GKSINKAVVTVPA---YFNDAQRQATK-DAGKIAGLDVQRIINEPTAAAL  227
 Os02g53420 KRQAV----TN-PQNTFFGTKRLIGRRFED--PQTQKEMKMVPYKIVK---APNG-DAWVETT--------DGKQYSPSQIGAFVLTKMKETAESYL---GKTVSKAVITVPA---YFNDAQRQATK-DAGRIAGLDVQRIINEPTAAAL  228
 Os03g02260 KRQAV----TN-PQNTFFGTKRLIGRRFDD--PQTQKEMKMVPYKIVK---ALNG-DAWLETT--------DGKQYSPSQIGAFVLTKMKETAESYL---GKSVSKAVITVPA---YFNDAQRQATK-DAGRIAGLDVQRIINEPTAAAL  228
 Bd3g57450 KRQAV----TN-PQNTFFGTKRMIGRRFDD--PQTQKEMKMVPYKIVK---APNG-DAWVETT--------DGKQYSPSQIGAFVLTKMKETAESYL---GKSISKAVITVPA---YFNDAQRQATK-DAGRIAGLDVQRIINEPTAAAL  228
 Bd1g69700 KEHAHEERDVL-SGSAIFNMKRLIGRMDTD---EVVQASKTLPFLVQT---LGIGVRPFIAALV-----NNMWRSTTPEEVLAIFLLELKALVEMHY---KHPVRNTILTIPV---AFSRFQQTRIE-RACAMAGLHVLRLMPEPTAVAL  211
 Os03g11910 REHGHEERDIL-SGSAIFNMKRLIGRMDTD---EVVQASKSLPFLVQT---LGIGVRPFIAALV-----NNMWRSTTPEEVLAIFLLELKALVEMHL---KHPVRNAVLTIPV---AFSRFQQTRIE-RACAMAGLHVLRLMPEPTAIAL  211
 AT2G32120 -----HEQEML-TGAAIFNMKRLVGRVDTD---PVVHASKNLPFLVQT---LDIGVRPFIAALV-----NNAWRSTTPEEVLAIFLVELRLMAEAQL---KRPVRNVVLTVPV---SFSRFQLTRFE-RACAMAGLHVLRLMPEPTAIAL  208
 Bd3g53100 AGIAA----RH-PSKVFARMRDLLGKPFPY--VQSLAQSLFLPYDFVQ---DARG-AAAVRADD-------G-QVYSVEEIVAMVLHYASGIADAHV---GAPVRDAVVAVPP---YFGQAERRSLT-QAAQLAGFNVLALINEHAGAAL  201
 Os02g48110 SGITA----RH-PSKVFARARDLLAKPFPY--VRSVAEALFLPYDLVP---DARG-AAAVRADD-------G-QVYTVEEIVAMVLHYAAGLADAHV---GAPVRDAVVAVPP---YFGQAERRALT-QAAQLAGVNVLALINEHAGAAL  209
 AT4G16660 AGITA----RY-PNKVYSQLRDMVGKPFKH--VKDFIDSVYLPFDIVE---DSRG-AVGIKIDD-------GSTVYSVEELLAMILGYASNLAEFHA---KIPVKDMVVSVPP---YFGQAERRGLI-QASQLAGVNVLSLVNEHSGAAL  204
 Bd1g32770 AGAAS----SHAPF---SSPKRLLLLAARP--ALVPRDLPRLPFPVHV---PADG-DALVHVDH-----IGRRIALSPTHLLAMLLAYLKQLAEADL---EAPVADCVISVPC---YFTQAHRRAYL-DAAAVAGLTPLRLMHDLAATAL  177
 Os06g46600 AGAAS----SHAPFSSIKRLLLLAGRPTLL--PRRGGDLSRLPFPVEASSADGGG-GVLVHVDH-----IGRRIALSPTQLLAMLLGYLRQLAEADL---EAPVSDCVISVPC---YFTQAQRQAYL-DAAAVAGLRPLRLMHDLAATAL  183
 AT1G11660 AASAT----MH-PKSTISQLKRLIGRKFRE--PDVQNDLRLFPFETSE---DSDG-GIQIRLRY-----MGEIQSFSPVQILGMLLSHLKQIAEKSL---KTPVSDCVIGIPS---YFTNSQRLAYL-DAAAIAGLRPLRLMHDSTATAL  179
 AT1G79920 AASTM----MN-PKNSISQIKRLIGRQFSD--PELQRDIKSLPFSVTE---GPDG-YPLIHANY-----LGEIRAFTPTQVMGMMLSNLKGIAEKNL---NTAVVDCCIGIPV---YFTDLQRRAVL-DAATIAGLHPLHLIHETTATAL  179
 AT1G79930 AASTM----MN-PKNSISQIKRLIGRQFSD--PELQRDIKSLPFSVTE---GPDG-YPLIHANY-----LGEKRAFTPTQVMGMMLSNLKGIAEKNL---NTAVVDCCIGIPV---YFTDLQRRAVL-DAATIAGLHPLRLIHETTATAL  179
 Os01g08560 AASST----MN-PKNSVSQIKRLLGRKYSD--PELQRDIAAFPFRVSE---GPDG-FPLVHARY-----LGEERVFTPTQLMAMVLSNLKGIAESNL---NTAVVDCCIGIPV---YFTDLQRRAVL-DAATIAGLCPLRLFHETTATAL  179
 Os05g08840 AASST----MN-PRNSVSQIKRLLGRAFAD--PELQRDLASFPFRVSE---GPDG-FPLVHARY-----LGEDRAFTPTQLLAMVLSNLKGIAEGNL---NAAVFDCCIGIPA---YFTDLQRRAVA-DAAAIAGLRPLRLFHETTATAL  179
     ruler .......160.......170.......180.......190.......200.......210.......220.......230.......240.......250.......260.......270.......280.......290.......300
```

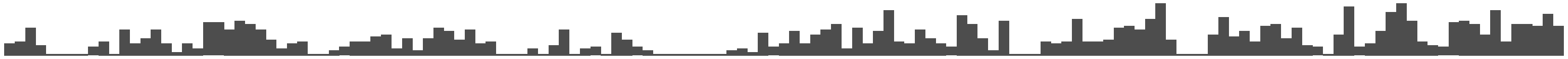

# CLUSTAL X (1.83) MULTIPLE SEQUENCE ALIGNMENT

**File: Z:Hsp reeA1.pep.ps** **Date: Tue Dec 18 15:07:21 2012**



```
            :.                    . .:*     .                                :** .:       :                        *    :     *:      : :            .   : * .:*
 AT5G28540 AYGLDKKGG---------EKNILVFDLGGGTFDVSVLTID------------NGVFEVLSTNGDTHLGGEDFDHRVMEYFIKLIKKKH--Q-KDISKDNKALGKLRRECERAKRALSSQHQVRVEIESLFDG----VDFSEPLTRARFEE   335
 AT5G42020 AYGLDKKGG---------EKNILVFDLGGGTFDVSVLTID------------NGVFEVLSTNGDTHLGGEDFDHRIMEYFIKLIKKKH--Q-KDISKDNKALGKLRRECERAKRALSSQHQVRVEIESLFDG----VDLSEPLTRARFEE   335
 Bd3g01480 AYGLDKKGG---------EKNILVFDLGGGTFDVSILTID------------NGVFEVLATNGDTHLGGEDFDHRIMEYFIKLIKKKY--S-KDISKDNRALGKLRREAERAKRALSNQHQVRVEIESLFDG----TDFSEPLTRARFEE   333
Os02g02410 AYGLDKKGG---------EKNILVFDLGGGTFDVSILTID------------NGVFEVLATNGDTHLGGEDFDQRIMEYFIKLIKKKY--S-KDISKDNRALGKLRREAERAKRALSNQHQVRVEIESLFDG----TDFSEPLTRARFEE   333
 AT1G09080 AYGLDKKGG---------ESNILVYDLGGGTFDVSILTID------------NGVFEVLSTSGDTHLGGEDFDHRVMDYFIKLVKKKY--N-KDISKDHKALGKLRRECELAKRSLSNQHQVRVEIESLFDG----VDFSEPLTRARFEE   349
Os03g50250 AYGLDRKGA-----G--EMTNVLVYDLGGGTFDVSVLSLD------------HGVFEVLATSGDTHLGGEDFDRRVMDHFIRLVKRQH--G-RDIGGDGRALGKLRRECERAKRALSRQHQVRVEIEALFVG----VDFSETLTRAKFEE   339
 Bd2g06050 AYGLDNKAK-----DAKEERNVLVFDLGGGTFDVSVLTID------------NGVFEVLATNGDTHLGGEDFDHRLMDYLVKLVKRKH--G-KDVSHDARALGKLRRECERAKRALSSQHQVRVEIESLFDG----VDLSEPLTRARFEE   348
Os05g35400 AYGIDKKGA---------EKNVLVFDLGGGTFDVSILAID------------NGVFEVLATNGDTHLGGEDFDQRLMDHFVKVIRRKH--G-RDIAGDARALGKLRRECERAKRALSNQHQVRVEIESLFDG----VDFSEPLSRARFEE   357
Os08g09770 AYGIDKKGA---------EKNVLVFDLGGGTFDVSILAID------------NGVFEVLATNGDTHLGGEDFDQRLMDHFVKVIRRKH--G-RDITGDARALGKLRRECERAKRALSNQHQVRVEVESLFDG----VDLSEPLSRARFEE   345
Os05g30480 AYGVGKEGA--------GGKNVLVFDLGGGTFDVSVLAID------------GGVYEVLATNGDTHLGGEDFDQRVMEHFVELVRRKH--G-RDIAGDARALGKLRRECERAKRALSIQHQVRVEVESLFDG----VDLSEPLSRARFEE   341
Os05g51360 AHGLHEDRG--------DGKVILVFHLGGRTAHATKFVIR------------DGTPSLIALRHDPFLGGDDFTARVVDHMADLIKDKH--GGRDVRADAAALRRLTAECERAKKALSYQQETVVTMRLDDD----DDLFSEPLTRSKLEE   345
Os06g10990 AYGHHTKQG--------DRKAILVFHLGGRTSHATIFKFV------------DGTARLIATRAHHFLGGDDFTARIVDHMVEHIKEQH--G-RDVRQEEKAMVRLRVACEHAKKALSEQQETLVQMDSLLDD---GAVFSATLTRAKFEE   366
 Bd4g43170 AHGLHRRL--------RNEGVALVLHVGGATTEASLMVLD------------DGVFDFLGGRHDAFLGGDDFDRRVVDYFTALMKRKH--G-RDISNDTQALAKLRTACEHAKKALSTRRQAEVVVESL--------GLAETLTRAEFEE   342
Os12g05760 SHGLHHGRL-------RDGGNVLVLHVGGGTSAATVLTYD------------NAVFEAVASRHDAHLGGDDFDARIAGRFSQLIKRDH--G-GG--VDDIAPAKLKSQCELAKRTLSSHDVAQVNLHATNL-ANGAFSFSGSLTRAQFEE   344
 Bd4g39820 AHGLHENEI---------TKNVLVLHVGGGTAEATVLVHE------------DGVFEAIGTWHDAHLGGDDFDRRTANHFLQLIREKH--S-VDIGNDTAKLWKLRAECERAKKELSDRDVARVSVNSIVD----GVDLFEPLTRAKFEE   336
 Bd4g39850 AHGLHENEI---------TKNVLVLHVGGGTAEATVLVHE------------DGVFEAIGTWHDAHLGGDDFDRRTANHFLQLIREKH--S-VDIGNDTAKLWKLRAECERAKKELSDRDVARVSVNSIVD----GVDLFEPLTRAKFEE   336
 AT4G24280 AYGFDRKAN----------ETILVFDLGGGTFDVSVLEVG------------DGVFEVLSTSGDTHLGGDDFDKRVVDWLAAEFKKDE--G-IDLLKDKQALQRLTEAAEKAKIELSSLTQTNMSLPFITATADGPKHIETTLTRAKFEE   378
 AT5G49910 AYGFERKSN----------ETILVFDLGGGTFDVSVLEVG------------DGVFEVLSTSGDTHLGGDDFDKRVVDWLASTFKKDE--G-IDLLKDKQALQRLTEAAEKAKIELSSLTQTNMSLPFITATADGPKHIETTLTRGKFEE   378
 Bd2g30560 AYGFEKKNN----------ETILVFDLGGGTFDVSVLEVG------------DGVFEVLSTSGDTHLGGDDFDKRIVDWLAGSFKNDE--G-IDLLKDKQALQRLTEAAEKAKMELSSLTQTNISLPFITATADGPKHIETTLTRAKFEE   347
Os05g23740 AYGFEKKNN----------ETILVFDLGGGTFDVSVLEVG------------DGVFEVLSTSGDTHLGGDDFDKRVVDWLAGNFKNDE--G-IDLLKDKQALQRLTEAAEKAKMELSSLTQTNISLPFITATADGPKHIETTLTRAKFEE   350
 Bd4g39470 AYGFEKKNN----------ETILVFDLGGGTFDVSVLEVG------------DGVFEVLSTSGDTHLGGDDFDKKIVDWLASTFKNDE--G-IDLLKDKQALQRLTEAAEKAKMELSTLTQANISLPFITATADGPKHIEATLSRAKFEE   350
Os12g14070 AYGFEKKNN----------ETILVFDLGGGTFDVSVLEVG------------DGVFEVLSTSGDTHLGGDDFDKKVVDWLASNFKKDE--G-IDLLKDKQALQRLTEAAEKAKMELSTLSQTNISLPFITATADGPKHIETTLSRAKFEE   361
 Bd4g33880 SYGMNNKE-----------GLIAVFDLGGGTFDVSILEIS------------NGVFEVKATNGDTFLGGEDFDAALLDYLISEYQKSD--N-IDLSKDKAVLQRLREAAEKAKVELSSTTQTEINLPFITADASGAKHFNITLTRSKFES   353
Os09g31486 SYGMNNKE-----------GLIAVFDLGGGTFDVSILEIS------------NGVFEVKATNGDTFLGGEDFDGALLDYLVSEFKKSD--N-IDLSKDKLALQRLREAAEKAKVELSSTMQTEINLPFITADATGAKHFNITLTRSKFES   354
 AT5G09590 SYGMTNKE-----------GLIAVFDLGGGTFDVSVLEIS------------NGVFEVKATNGDTFLGGEDFDNALLDFLVNEFKTTE--G-IDLAKDRLALQRLREAAEKAKIELSSTSQTEINLPFITADASGAKHFNITLTRSRFET   356
 AT4G37910 SYGMNNKE-----------GVIAVFDLGGGTFDVSILEIS------------SGVFEVKATNGDTFLGGEDFDNTLLEYLVNEFKRSD--N-IDLTKDNLALQRLREAAEKAKIELSSTTQTEINLPFITADASGAKHLNITLTRSKFEG   351
Os02g53420 SYGTNNKE-----------GLIAVFDLGGGTFDVSILEIS------------NGVFEVKATNGDTFLGGEDFDNTLLEFLVSEFKRSE--A-IDLAKDRLALQRLREAAEKAKIELSSTAQTEINLPFITADASGAKHLNITLTRSKFES   352
Os03g02260 SYGTNNKE-----------GLIAVFDLGGGTFDVSILEIS------------NGVFEVKATNGDTFLGGEDFDNTLLEFLVSEFKRTE--G-IDLSKDRLALQRLREAAEKAKIELSSTAQTEINLPFITADSSGAKHLNITLTRSKFES   352
 Bd3g57450 SYGTNNKE-----------GLIAVFDLGGGTFDVSILEIS------------NGVFEVKATNGDTFLGGEDFDNTLLEFLVSEFKRTD--A-IDLSKDRLALQRLREAAEKAKIELSSTAQTEINLPFITADASGAKHLNITLTRSKFES   352
 Bd1g69700 LYAQQQQQLLHDNMGSGIEKIALIFNMGAGYCDVAVSATA------------GGVSQIRALSGCT-VGGEDILQNVMRHLHPNFDSLF--A-GHTMDRIKSMGLLRIATQDAVHKLTTQESIEINVDLGGG-----QKVSKVLGRAEFEQ   340
Os03g11910 LYAQQQQQLLHDNMGSGIEKIALIFNMGAGYCDAAVAATA------------GGVSQIRALSGST-VGGEDILQNVMRHLMPDFDSLY--A-GQTMDRIKSIGLLRMATQDAIHKLATQEHVEINVDLGAG-----HKVSKILDRGEFEK   340
 AT2G32120 LYAQQQQMTTHDNMGSGSERLAVIFNMGAGYCDVAVTATA------------GGVSQIKALAGSP-IGGEDILQNTIRHIAPPNEE--------------ASGLLRVAAQDAIHRLTDQENVQIEVDLGNG-----NKISKVLDRLEFEE   326
 Bd3g53100 QYGIDKDFS----NG---SRHVIFYDMGSGSTYAALVYYSSYNAKEFGKTVSVNQFQVKDVRWNSKLGGIEMEMRLVNYFADQFNKQLGNG-DDIRQSPKAMAKLKKQVKRTKEILSANTAAPISVESLYND----IDFRSTITREKFEE   339
Os02g48110 QYGIDKDFS----NE---SRHVIFYDMGAGSTYAALVYYSAYKAKEFGKTVSVNQFQVKDVRWDSKLGGLEMEMRLVNYFADQFNKQLGNG-VDIRQSPKAMAKLKKQVKRTKEILSANTAAPISVESLYND----LDFRSTITREKFEE   347
 AT4G16660 QYGIDKDFA----NG---SRHVIFYDMGSSSTYAALVYYSAYSEKEYGKTVSVNQFQVKDVRWDLGLGGQSMEMRLVEHFADEFNKQLGNG-VDVRKFPKAMAKLKKQVKRTKEILSANTAAPISVESLHDD----RDFRSTITREKFEE   342
 Bd1g32770 GYGLYRSDL----GVAGSPTFVAFVDVGHSDTQATVVAFD------------PSGMKVLSHGFDADLGGRDFDEVLFEHFAEEFRDRY--K-IDVVGNVKASMRLRAACEKAKKVLSANAEAVVNIECLMEE----KDVRGMIRREEFEK   304
Os06g46600 GYGLYRSDL----GGPGGPTYVAFVDVGHCDTQVAVVAFD------------VSGMKVLSHRFDADLGGRDFDEVLFEHFAEEFRDKY--K-IDVTGNVKASMRLRAACEKAKKVLSANAEAVVNIECLMEE----KDVRGMIRREEFEK   310
 AT1G11660 GYGIYKTDL----VANSSPTYIVFIDIGHCDTQVCVASFE------------SGSMRVRSHAFDRNLGGRDFDEVLFNHFALEFKEKY--N-IDVYTNTKACVRLRASCEKVKKVLSANAEAQLNIECLMEE----KDVRSFIKREEFEQ   306
 AT1G79920 AYGIYKTDL----PEND-QLNVAFIDIGHASMQVCIAGFK------------KGQLKILSHAFDRSLGGRDFDEVLFNHFAAKFKDEY--K-IDVSQNAKASLRLRATCEKLKKVLSANPMAPLNIECLMAE----KDVRGVIKREEFEE   305
 AT1G79930 AYGIYKTDL----PESD-QLNVAFIDIGHASMQVCIAGFK------------KGQLKILSHAFDRSLGGRDFDEVLFNHFAAKFKDEY--K-IDVSQNAKASLRLRATCEKLKKVLSANPLAPLNIECLMDE----KDVRGVIKREEFEE   305
Os01g08560 AYGIYKTDL----PEND-QLNVAFVDVGHASMQVCIAGYK------------KGQLKILSHAYDRSLGGRDFDEVLFKHFAAKFKDEY--K-IDVYQNARACIRLRVACEKLKKVLSANPESPMHIECLMDE----KDVRGFIKREEFEK   305
Os05g08840 AYGIYKTDL----PEKE-WLNVAFIDVGHASMQVSIVGYK------------KGQLNMLSHAYDRSLGGRDFDEVLFKHFAEKFKDEY--K-IDVYQNARACVRLRVACEKLKKMLSANPEAPLNIECLMDE----KDVRGFIKREEFEQ   305
     ruler .......310.......320.......330.......340.......350.......360.......370.......380.......390.......400.......410.......420.......430.......440.......450
```

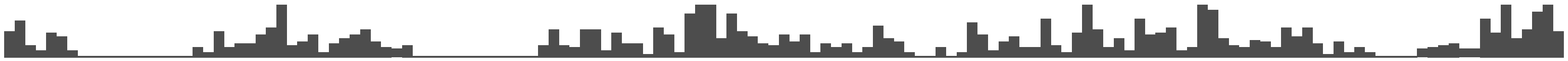

# CLUSTAL X (1.83) MULTIPLE SEQUENCE ALIGNMENT

**File: Z:Hsp reeA1.pep.ps** **Date: Tue Dec 18 15:07:21 2012**



```
            :   :  .                        :  : ::*. : :* :   :                          . :    *  :
AT5G28540  LNNDLFRKTMGPVKKAMDDAGLQK------SQIDEIVLVGGSTRIPKVQQLLKDFFEGKEPN------------KGVNPDEAVAYGAAVQGGILSGEGGD-ETKDILLLDVAPLTLGIETVG-----G-------VMTKLIPRNTVIPTK  454
AT5G42020  LNNDLFRKTMGPVKKAMDDAGLQK------SQIDEIVLVGGSTRIPKVQQLLKDFFEGKEPN------------KGVNPDEAVAYGAAVQGGILSGEGGD-ETKDILLLDVAPLTLGIETVG-----G-------VMTKLIPRNTVIPTK  454
Bd3g01480  LNNDLFRKTMGPVKKAMDDAGLEK------SQIHEIVLVGGSTRIPKVQQLLRDYFEGKEPN------------KGVNPDEAVAFGAAVQGSILSGEGGD-ETKDILLLDVAPLTLGIETVG-----G-------VMTKLIPRNTVIPTK  452
Os02g02410  LNNDLFRKTMGPVKKAMDDAGLEK------SQIHEIVLVGGSTRIPKVQQLLRDYFEGKEPN------------KGVNPDEAVAYGAAVQGSILSGEGGD-ETKDILLLDVAPLTLGIETVG-----G-------VMTKLIPRNTVIPTK  452
AT1G09080  LNMDLFKKTMEPVKKALKDAGLKK------SDIDEIVLVGGSTRIPKVQQMLKDFFDGKEPS------------KGTNPDEAVAYGAAVQGGVLSGEGGE-ETQNILLLDVAPLSLGIETVG-----G-------VMTNIIPRNTVIPTK  468
Os03g50250  LNMDLFKKTLGPVRKAIADAKLKK------SDIDEIVLVGGSTRIPKVQELLKEMFDGKEPT------------KGINPDEAVAYGAAVQGSIISGEGGA-ETKDILLLDVTPLTLGIETAG-----G-------VMTKLIPRNTRIPVK  458
Bd2g06050  LNSDLFRKTMTPVKKAMADAGLAK------GDIHEVVLVGGSTRIPKIQQFLKDYFDGKEPS------------KGVNPDEAVAYGAAVQGSIVRGD----NAEKLVVLDVTPLTLGIETAG-----G-------VMTPLIPRGTVIPTR  464
Os05g35400  LNGDLFKKTMVPVRKAMADAGLGK------GDIDEIVLVGGSTRIPKVQQLLKDYFGGKEPN------------RGVNPDEAVAYGAAVQASIISGHVDE-NTESMILLDVAPLTLGLETAG-----G-------VMTKLIPRNTVVPTK  476
Os08g09770  LNSDLFKKTMVPVRKAMADARLSK------GDIDEIVLVGGSTRIPKVQQLLKDYFGGKEPN------------RGVNPDEAVAYGAAVQASIISGHVDE-NTESMILLDVAPLTLGLETAG-----G-------VMAKLIPRNTVVPTK  464
Os05g30480  LNNDLFRKTMAPVRKAMADARLSN------ADIDEIVLVGGSTRIPKVRQLLRDYFGGKQPN------------QGVNPDEAVAYGAAIQANIVGGDTDN-KTRDMVVLDVTPLTLGLETAG-----G-------VMATLIPRNTPVPTK  460
Os05g51360  LIGDLVGRAVDLVESCDASGGDVV-------GVDEILLVGGSTRIPMVRDLVKDYFHGKEAS----------NEKGVEPDEAVIRGALLLSHPHQARYLD-P---------------------------------CYDYWHSR-------  437
Os06g10990  LNHDLLDRAMALVKEVVVTTGGVE-------VVDEVLVVGGSARIPKVRQLVKDYFNGNGNG-THPNSRGCKGPVDVEPEDAVLHGAALLSRPLPVAEGT-A---------------------------------AARSIGSVGGI----  470
Bd4g43170  LNGDLFGEVVKLVHRAMVGADRELDGRSIWDAVDEVLLVGGSAVIPEIQRLVRDYFGGRKKVAVH---------AGVKPDEVVTLGGALLTRADAGG--------------------YPCMG-----V------DGRRQRGYHSDWCDNR  452
Os12g05760  LNHDLFEKVISLVDAAMAEARRAVAG---FDVIDEVVLVGGSTKIPRIRELIKNYFAGKEATVKATASIGGGAVVVVEPEEAVVHGGGLLSHPMEDG--------------------YHCMG-----A------GGRRQIGSPMDRCYHE  460
Bd4g39820  LNHDLFGRIMSVVDKAMVGSELAE-K---KELVDEIVLVGGSSRMPRIQELISDYFGGRQVRIGT---------AAVEPDEAVVHGGALLSHPTAGG--------------------YECLG-----G-------ERRQFGASTDLC---  438
Bd4g39850  LNHDLFGRIMSVVDKAMVGSELAE-K---KELVDEIVLVGGSSRMPRIQELISDYFGGRQVRIGT---------AAVEPDEAVVHGGALLSHPTAGG--------------------YECLG-----G-------ERRQFGASTDLC---  438
AT4G24280  LCSDLLDRVRTPVENSLRDAKLSF------KDIDEVILVGGSTRIPAVQELVRKVT-GKEPN------------VTVNPDEVVALGAAVQAGVLAGD-----VSDIVLLDVTPLSIGLETLG-----G-------VMTKIIPRNTTLPTS  492
AT5G49910  LCSDLLDRVRTPVENSLRDAKLSF------KDIDEVILVGGSTRIPAVQDLVRKLT-GKEPN------------VSVNPDEVVALGAAVQAGVLSGD-----VSDIVLLDVTPLSLGLETLG-----G-------VMTKIIPRNTTLPTS  492
Bd2g30560  LCSDLLDRLRTPVDNSLRDAKLSL------KEIDEVILVGGSTRIPAVQDLVKKMT-GKDPN------------VTVNPDEVVALGAAVQAGVLSGD-----VSDIVLLDVTPLSLGLETLG-----G-------VMTKIIPRNTTLPTS  461
Os05g23740  LCSDLLDRLRTPVDNALRDAKLSF------KDIDEVILVGGSTRIPAVQDLVKKMT-GKDPN------------VTVNPDEVVALGAAVQAGVLSGD-----VSDIVLLDVTPLSLGLETLG-----G-------VMTKIIPRNTTLPTS  464
Bd4g39470  LCSDLIDRLKTPVNNALKDAKLSV------SNLDEVILVGGSTRIPSVQELVKKIT-GKDPN------------VTVNPDEVVSLGAAVQGGVLAGD-----VKDVVLLDVTPLSIGLETLG-----G-------VMTKIIPRNTTLPTS  464
Os12g14070  LCSDLIDRLKTPVTNALRDAKLSV------DNLDEVILVGGSTRIPSVQELVKKIT-GKDPN------------VTVNPDEVVSLGAAVQGGVLAGD-----VKDVVLLDVTPLSLGLETLG-----G-------VMTKIIPRNTTLPTS  475
Bd4g33880  LVGNLIERTRIPCVNCLKDAGISA------KEIDEVLLVGGMTRVPKVQDIVSQKF-NKAPS------------KGVNPDEAVAMGAAIQGGILRGD-----VKELLLLDVTPLSLGIETLG-----G-------IFTRLINRNTTIPTK  467
Os09g31486  LVQSLIERTRIPCVNCLKDAGVSA------KDIDEVLLVGGMTRVPKVQDIVSQIF-NKTPS------------KGVNPDEAVAMGAAIQGGILRGD-----VKELLLLDVTPLSLGIETLG-----G-------IFTRLINRNTTIPTK  468
AT5G09590  LVNHLIERTRDPCKNCLKDAGISA------KEVDEVLLVGGMTRVPKVQSIVAEIF-GKSPS------------KGVNPDEAVAMGAALQGGILRGD-----VKELLLLDVTPLSLGIETLG-----G-------VFTRLITRNTTIPTK  470
AT4G37910  LVGKLIERTRSPCQNCLKDAGVTI------KEVDEVLLVGGMTRVPKVQEIVSEIF-GKSPC------------KGVNPDEAVAMGAAIQGGILRGD-----VKDLLLLDVVPLSLGIETLG-----A-------VFTKLIPRNTTIPTK  465
Os02g53420  LVNSLIERTREPCKNCLKDAGITT------KEVDEVLLVGGMTRVPKVQEIVSEIF-GKSPS------------KGVNPDEAVAMGAAIQGGILRGD-----VKELLLLDVTPLSLGIETLG-----G-------IFTRLINRNTTIPTK  466
Os03g02260  LVNSLIERTRDPCKSCLKDAGITT------KDVDEVLLVGGMTRVPKVQEVVSEIF-GKAPS------------KGVNPDEAVAMGAAIQGGILRGD-----VKDLLLLDVTPLSLGIETLG-----G-------IFTRLINRNTTVPTK  466
Bd3g57450  LVSGLIARTRDPCKNCLKDAGITT------KEVDEVLLVGGMTRVPKVQEVVSEIF-GKAPS------------KGVNPDEAVAMGAALQGGILRGD-----VKELLLLDVTPLSLGIETLG-----G-------IFTRLISRNTTIPTK  466
Bd1g69700  VNKMIFEECERVIKQCLHDAKLAP------EDINDVILVGGCSRIPKIRSLVLGLCKKADSY------------ASIDVLEAAVSGAALEGAIASGVTDPSGSLDLLTIQATPMNLGIRADG-----D-------TFAAIIPRNTAVPAR  460
Os03g11910  VNQSIFEKCEGIIKQCLADAKLTP------EDINDVILVGGCSRIPKIRSVVLGLCKKDDSY------------SGIDPLEAAVSGAALEGAIASGVSDPSGSLDLLTIQATPMNLGIRADG-----G-------NFAAIIPRNTAVPAR  460
AT2G32120  VNQKVFEECERLVVQCLRDARVNG------GDIDDLIMVGGCSYIPKVRTIIKNVCKKDEIY------------KGVNPLEAAVRGAALEGAVTSGIHDPFGSLDLLTIQATPLAVGVRANG-----N-------KFIPVIPRNTMVPAR  446
Bd3g53100  LCEDLWEQALTPIKEVLVQSGMKI------DDIYAVELIGGATRVPKLQAKLQEFLGRSELD------------KHLDADEAIVLGASLHAANLSDGIKL--NRKLGMIDGSPYGFVFEIN-----GPDYVKDESTDQVLVPRMKKMPIK  464
Os02g48110  LCEELWEQALTPVKEVLAHSGMKI------DDIYAVELIGGATRVPKLQAKLQEFLGRSDLD------------KHLDADEAIVLGASLHAANLSDGIKL--NRKLGMIDGSTYGFVFEIN-----GPDYVKDESTDQLLVPRMKKLGIK  472
AT4G16660  LCKDLWERSLTPLKDVLKHSGLKI------DDISAVELIGGATRVPKLQSTIQEFIGKQQLD------------KHLDADEAIVLGSALHAANLSDGIKL--KRRLGIVDGSPYGFLVELE-----GPNVKKDESTKQQLVPRMKKLPSK  467
Bd1g32770  LCSQLLERVVEPCKRAMADSGVGL------EKLQSVELVGSGSRVPAIARVLAEFFRR-EPS------------RTINVSECVARGCALQCAMLSPTLRV---REYEVQDAIPASIGFCTN-------EGPISTLSSNALFRRGQPLPSV  425
Os06g46600  LCAGLLERVVEPCKKAMEGSRIGF------DRLHSVELVGSGSRVPAIARILAGFFRR-EPS------------RTLNASECVARGCALQCAMLSPTFRV---REYEVQDAIPSSIGFCTS-------EGPISTLPSNALFQRGHPLPSV  431
AT1G11660  LSAGLLERLIVPCQKALADSGLSL------DQIHSVELVGSGSRIPAISKMLSSLFKR-ELG------------RTVNASECVARGCALQCAMLSPVFRV---RDYEVQDSYPFAIGFSSD-------KGPINTPSNELLFPKGQIFPSV  427
AT1G79920  ISIPILERVKRPLEKALSDAGLTV------EDVHMVEVVGSGSRVPAMIKILTEFFGK-EPR------------RTMNASECVSRGCALQCAILSPTFKV---REFQVHESFPFSISLAWKGAATDAQNGGTENQQSTIVFPKGNPIPSV  433
AT1G79930  ISIPILERVKRPLEKALSDAGLTV------EDVHMVEVIGSGSRVPAMIKILTEFFGK-EPR------------RTMNASECVSRGCALQCAILSPTFKV---REFQVHESFPFSISLAWKGAASEAQNGGAENQQSTIVFPKGNPIPSV  433
Os01g08560  ISASILERVKGPLEKALAEAGLTT------ENVHFVEVVGSGSRVPAIIKILTDFFGK-EPR------------RTMNASECVARGCALECAILSPTFKV---REFQVNDGFPFSIAMSWKPDSQNGD------NQQTVVFPKGNPLPSV  427
Os05g08840  ISSPVLQRVKAPLEKALAEAGLTT------ENVHFVEVVGSGSRVPAIIRIITEFFGK-EPR------------RTMNASECVARGCALQCAVLSPTFKV---REFEVNDGFPFSIALSCKPDSENTE------SEQTIVFPKGSPVPSA  427
     ruler .......460.......470.......480.......490.......500.......510.......520.......530.......540.......550.......560.......570.......580.......590.......600
```

```
AT5G28540  KSQVFTTYQDQQTTVSIQVFEGERSLT--KDCRLLGKFDLNGIPPAPRGT--PQIEVTFEVDANGILNVKAEDKASGKSEKIT-------------------------------------------------------------------  533
AT5G42020  KSQVFTTYQDQQTTVSIQVFEGERSLT--KDCRLLGKFDLTGVPPAPRGT--PQIEVTFEVDANGILNVKAEDKASGKSEKIT-------------------------------------------------------------------  533
Bd3g01480  KSQVFTTYQDQQTTVSIQVFEGERSMT--KDCRLLGKFDLSGIPPAPRGT--PQIEVTFEVDANGILNVKAEDKGTGKSEKIT-------------------------------------------------------------------  531
Os02g02410 KSQVFTTYQDQQTTVSIQVFEGERSMT--KDCRLLGKFDLSGIPAAPRGT--PQIEVTFEVDANGILNVKAEDKGTGKSEKIT-------------------------------------------------------------------  531
AT1G09080  KSQVFTTYQDQQTTVTINVYEGERSMT--KDNRELGKFDLTGILPAPRGV--PQIEVTFEVDANGILQVKAEDKVAKTSQSIT-------------------------------------------------------------------  547
Os03g50250 KSQVFTTYEDHQTTVSIKVFEGERSLT--KDCRELGRFDLSGIAPAPRGV--PQIEVTFEVDENGILHVTASDKAAGRSKSIT-------------------------------------------------------------------  537
Bd2g06050  KTKTFTTYQDRQTTVSVVVFEGERSMT--KDNKQLGKFDLTGIAPAPRGT--PQIEVTFEVDVNGILHVKAADKGTGKSEKIQ-------------------------------------------------------------------  543
Os05g35400 KTQVFTTYKDRQTTVTIQVFEGERSMT--RDNRLLGKFDLTGIAPAPRGA--PQIAVTFEVDANGILSVLAADKATGRSEKIT-------------------------------------------------------------------  555
Os08g09770 KTQVFTTYKDKQTTVTIQVFEGERSMT--RDNRLLGRFDLAGIAPAPRGA--PQIEVTFEVDANGILSVLAADKATGRSEKIT-------------------------------------------------------------------  543
Os05g30480 RAQLFSTYKDKQTTVTVKVFEGERSMT--RDNRLLGRFDLAGIAPAPRGA--PQIEVAFEVDADGILSVSAADRATGRSERIT-------------------------------------------------------------------  539
Os05g51360 ------------------------------------------------------------------------------------------------------------------------------------------------------  437
Os06g10990 ------------------------------------------------------------------------------------------------------------------------------------------------------  470
Bd4g43170  RW----------------------------------------------------------------------------------------------------------------------------------------------------  454
Os12g05760 F-----------------------------------------------------------------------------------------------------------------------------------------------------  461
Bd4g39820  ------------------------------------------------------------------------------------------------------------------------------------------------------  438
Bd4g39850  ------------------------------------------------------------------------------------------------------------------------------------------------------  438
AT4G24280  KSEVFSTAADGQTSVEINVLQGEREFV--RDNKSLGSFRLDGIPPAPRGV--PQIEVKFDIDANGILSVSAVDKGTGKKQDIT-------------------------------------------------------------------  571
AT5G49910  KSEVFSTAADGQTSVEINVLQGEREFV--RDNKSIGSFRLDGIPPAPRGV--PQIEVKFDIDANGILSVSASDKGTGKKQDIT-------------------------------------------------------------------  571
Bd2g30560  KSEVFSTAADGQTSVEINVLQGEREFV--RDNKSLGSFRLDGIPPAPRGV--PQIEVKFDIDANGILSVAAVDKGTGKKQDIT-------------------------------------------------------------------  540
Os05g23740 KSEVFSTAADGQTSVEINVLQGEREFV--RDNKSLGSFRLDGIPPAPRGV--PQIEVKFDIDANGILSVSAVDKGTGKKQDIT-------------------------------------------------------------------  543
Bd4g39470  KSEVFSTAADGQTSVEINVLQGEREFV--RDNKSLGSFRLDGIPPAPRGV--PQIEVKFDIDANGILSVAAVDKGTGKKQDIT-------------------------------------------------------------------  543
Os12g14070 KSEVFSTAADGQTSVEINVLQGEREFV--RDNKSLGSFRLDGIPPAPRGV--PQIEVKFDIDANGILSVAAIDKGTGKKQDIT-------------------------------------------------------------------  554
Bd4g33880  KSQTFSTAADNQTQVGIKVLQGEREMA--TDNKLLGEFQLEGIPPAPRGM--PQIEVTFDIDANGIVRVSAKDKSTGKEQDIT-------------------------------------------------------------------  546
Os09g31486 KSQVFSTAADNQTQVGIKVLQGEREMA--TDNKLLGEFQLEGIPPAPRGM--PQIEVTFDIDANGIVKVSAKDKSTGKEQEIT-------------------------------------------------------------------  547
AT5G09590  KSQVFSTAADNQTQVGIRVLQGEREMA--TDNKLLGEFDLVGIPPSPRGV--PQIEVTFDIDANGIVTVSAKDKTTGKVQQIT-------------------------------------------------------------------  549
AT4G37910  KSQVFSTAADNQMQVGIKVLQGEREMA--ADNKVLGEFDLVGIPPAPRGM--PQIEVTFDIDANGITTVSAKDKATGKEQNIT-------------------------------------------------------------------  544
Os02g53420 KSQVFSTAADNQTQVGIRVLQGEREMA--TDNKLLGEFDLVGIPPAPRGM--PQIEVTFDIDANGIVTVSAKDKSTGKEQQIT-------------------------------------------------------------------  545
Os03g02260 KSQVFSTAADNQTQVGIKVLQGEREMA--ADNKLLGEFDLVGIPPAPRGM--PQIEVTFDIDANGIVTVSAKDKATGKEQQIT-------------------------------------------------------------------  545
Bd3g57450  KSQVFSTAADNQTQVGIRVLQGEREMA--TDNKLLGEFDLVGIPPAPRGL--PQIEVTFDIDANGIVTVSAKDKATAKEQQIT-------------------------------------------------------------------  545
Bd1g69700  RDMLFTTTHDNQTEALVAVYEGEGKQA--EENHLLGYFKITGIPAAPKGA--VEINVCMDIDAANVLRVFAGVVKPQGPA-TPPFI----------------------------------------------------------------  541
Os03g11910 RDMLFTTTHDNQTEALIAVYEGEGNQA--EDNHLLGYFKITGIPAAAKGT--VEINVCMDIDAGNVLRVFAGVVKPQGQA-VPPFI----------------------------------------------------------------  541
AT2G32120  KDLFFTTVQDNQKEALIIIYEGEGETV--EENHLLGYFKLVGIPPAPKGV--PEINVCMDIDASNALRVFAAVLMPGSSSPVVPVI----------------------------------------------------------------  528
Bd3g53100  LYRSVKHTKDFDVSL---SYDKASELPPGVSSHKFAEYSISGLADASEKYGSRNLSAPIKANLHFSLSRSGIISLDRAEAVIEITEWAEIPKKNLTLESNATDQTLSSESGTS-DSTADSKEN--PSSGSDANNLSNTN-DEGNVRDAIT  607
Os02g48110 MFRSIRHTKDFDVSI---SYEKASELPPGVTSHKFVEYSVSGLTDASEKYSSRNLSAPIKANLHFSLSRSGIISLDRAEAVIEITEWVEVPKKNLTLESNSTSQTLSSEGGAA-NDTSDSKEN--VSSDGDANKSSAPI-DESNAQDIVT  615
AT4G16660  MFRSFVLDKDFDVSL---AYESEGILPPGTTSPVFAQYSVSGLADASEKYSSRNLSAPIKANLHFSLSRSGILSLDRGDAVIEITEWVDVPKKNVTIDSNTTTST---GNATD-ENSQENKED--LQTDAENSTASNTTAEEPAVASLGT  608
Bd1g32770  KIITLHRNSGFNLDV---YYLDENELPPGT-STKIGSFQIGPFHAH-TEK--SKVKVKIRLNLHGLISVESAVLIEDDQRDTNSS-------DSMEVDHNND---------------------------------------------VGD  516
Os06g46600 KVVTLHKNSKFKLDA---FYVDENELPPGT-STKIGAFQIGPFQAH-TEK--SKVKVRIRLNLHGLVSVESAALIDDDQSDAHSA-------DSMEVDSNGE---------------------------------------------MGQ  522
AT1G11660  KVLTLHRENTFQLEA---FYANHNELSPDI-PTQISSFMIGPFHISHGEA--ARVKVRVQLNLHGIVTIDSATLIEYHKENITSE-------EMISEENHQSSA----------------------------------MKDGSLDPSSGS  530
AT1G79920  KALTFYRSGTFSIDV---QYSDVNDLQA---PPKISTYTIGPFQSSKGER--AKLKVKVRLNLHGIVSVESATLLEEEEVEVSVT--KDQSEETAKMDTDKASAEAAPASGDSDVNMQDAKDTSDA--TGTDNGVPESAEKPVQMETDSK  571
AT1G79930  KALTFYRSGTFSVDV---QYSDVNDLQA---PPKISTYTIGPFQSSKGER--AKLKVKVRLNLHGIVSVESATLLEEEEVEVPVT--KEHSEETTKMDSDKASAEAAPASGDCDVNMQDAKDTSDA--TGTDNGVPESAEKPVQMETDSK  571
Os01g08560 KALTFYRSNTFQVDV---TYVDTGDLQI---SPKISTYTVGPFNPGKGDK--AKLKVKVRLNIHGVVTVESATMLEEEEVEVPVAATTEPPKDSAKMETDDAPNEAASG---TDVNMQEAKAPADAAADGAENGAPNSEEKSVPMETDAK  566
Os05g08840 KTVTFYRSNTFAVDV---VSVDADDLQM---AKKISSYTIGPFQSSKPEK--AKVNVKACLNIHGIVSIESAMMLEE-EVDVPVATTNETLKDDTKMDTDDALGDPASG---TDENMQESKCSADATHGAAENGKPDSEEISAPMDTDAK  565
ruler      .......610.......620.......630.......640.......650.......660.......670.......680.......690.......700.......710.......720.......730.......740.......750
```

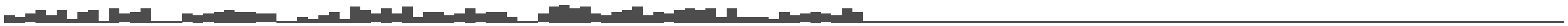

# CLUSTAL X (1.83) MULTIPLE SEQUENCE ALIGNMENT

**File: Z:Hsp reeA1.pep.ps** **Date: Tue Dec 18 15:07:21 2012**



```
AT5G28540 -----------------IT-NEKGRLSQEEIDRMVKEAEEFAEEDKKVKEKIDARNALETYVYNMKNQVNDKDKLADKLEGDEKEKIEAATKEALEWLDENQ----NSEKEEYDEKLKEVEAVCNPIITAVYQRSGGAPGGAGGESST-- 659
AT5G42020 -----------------IT-NEKGRLSQEEIDRMVKEAEEFAEEDKKVKEKIDARNALETYVYNMKNQVSDKDKLADKLEGDEKEKIEAATKEALEWLDENQ----NSEKEEYDEKLKEVEAVCNPIITAVYQRSGGAPGAGGESS-T-- 658
Bd3g01480 -----------------IT-NEKGRLSQEEIDRMVKEAEEFAEEDKKVKERIDARNQLETYVYNMKNTVGDKDKLADKLESEEKEKVEEALKEALEWLDENQ----SAEKEDYEEKLKEVEAVCNPIVSAVYQRSGGAPGGEDGAGGV-- 657
Os02g02410 -----------------IT-NEKGRLSQEEIDRMVREAEEFAEEDKKVKERIDARNQLETYVYNMKNTVGDKDKLADKLESEEKEKVEEALKEALEWLDENQ----TAEKEEYEEKLKEVEAVCNPIISAVYQRTGGAPGGGADGEGG-- 657
AT1G09080 -----------------IT-NDKGRLTEEEIEEMIREAEEFAEEDKIMKEKIDARNKLETYVYNMKSTVADKEKLAKKISDEDKEKMEGVLKEALEWLEENV----NAEKEDYDEKLKEVELVCDPVIKSVYEKTEGENEDDDGDDHD-- 673
Os03g50250 -----------------IT-NDKGRLSQEEIDRMVREAEEFAEEDRRVRERVDARNRLENYVYRMRSAVRDG-GMAGKIGDDDRERMESALTEALEWLEDNDGGARTAEKEDYEEKLKEVEQVCGPIIKQVYKKSGDASAGAGDDDDV-- 666
Bd2g06050 -----------------ITSAADRRITQEEIDRMVREAEEFAEEDRKVRERVDARNRMEAYVYHVRTTV-DG-EAGQGMDGGDKERVREAAREASEWIDENP----EADKDEYVEKLKELEDLCNPVFAAADSHKSGGGHDEAE------ 664
Os05g35400 -----------------I-SGDDRKISQEEIDRMVREAEEFADEDRRHREQVDARNSLEAYVYNVKSTL-GG-KMADAMEGEEKEKVEEAVREAHEWLDGNP----DAGKEEYEEKLRELEDVCNPVMSAVYQRSGGGGGAP-EDGNV-- 678
Os08g09770 -----------------I-SGDDRKISQEEIDRMVREAEEFAEEDRRHREQVDARNSLEAYVYNIKNTL-GG-KMADAMEGEEKDKVEEAVREAYEWLDGNP----DAGKEEYEEKLRELEDVCNPVMSAVYQRSGGGGGGAPEDGNV-- 667
Os05g30480 -----------------I-SGDDRKTSREEIDRMLGEAEEFADEDRRHRERAGARNSLEAYVYGVKNAVVGG-EMAGAMDGGEKEKVEAAVMEAYEWLDGNQ----DVGKEEYEEKLRELEDVCNPVMSAVYQRSGGSRRDGDG------ 660
Os05g51360 ------------------------------------------------------------------------------------------------------------------------------------------------------ 437
Os06g10990 ------------------------------------------------------------------------------------------------------------------------------------------------------ 470
Bd4g43170 ------------------------------------------------------------------------------------------------------------------------------------------------------ 454
Os12g05760 ------------------------------------------------------------------------------------------------------------------------------------------------------ 461
Bd4g39820 ------------------------------------------------------------------------------------------------------------------------------------------------------ 438
Bd4g39850 ------------------------------------------------------------------------------------------------------------------------------------------------------ 438
AT4G24280 -----------------IT--GASTLPKDEVDQMVQEAERFAKDDKEKRDAIDTKNQADSVVYQTEKQLK---ELGEKIPGEVKEKVEAKLQELKDKIGSGS-------TQEIKDAMAALNQEVMQIGQSLYNQPGAGGPGAGPSPGGEG 692
AT5G49910 -----------------IT--GASTLPKDEVDTMVQEAERFAKEDKEKRDAIDTKNQADSVVYQTEKQLK---ELGEKIPGPVKEKVEAKLQELKEKIASGS-------TQEIKDTMAALNQEVMQIGQSLYNQPQPGGADSPPGGEASS 692
Bd2g30560 -----------------IT--GASTLPKDEVEKMVEEAEKFAAEDKEKRDAIDTKNQAESVIYQTEKQLK---ELGDKVPGEVKGKVEGKLVELKDAVAGGT-------TQTIKDALAALNQEVMQLGQSLYQQQGAPGAGPTPGGDGTA 661
Os05g23740 -----------------IT--GASTLPKDEVEKMVEEAEKFAKEDKEKRDAIDTKNQAESVIYQTEKQLK---ELGDKVPGDVKGKVEAKLTELKDAVAGGS-------TQTMKDALAALNQEVMQLGQALYSQQGAPGAGPTPGADAAA 664
Bd4g39470 -----------------IT--GASTLPKDEVERMVEEADKFAQEDKEKRDAIDTKNQADSVVYQTEKQLK---ELGDKVPAPVKEKVDVKLQELKDAIAGGS-------TPTMKAAMEALNQEVMQIGQAMYNQSSGGASGPTDAGA--E 662
Os12g14070 -----------------IT--GASTLPKDEVERMVEEADKFAQEDKEKRDAIDTKNQADSVVYQTEKQLK---ELGDKVPAPVKEKVDAKLNELKEAIAGGS-------TQSMKDAMAALNEEVMQIGQAMYNQQPNAGAAGPTPGA--D 673
Bd4g33880 -----------------IK--SSGGLSERDIENMVKEAELHSQRDQEKKSLIDLKNSADTTIYSIEKSVS---EYKDKVPSEITTEVESAVSDLRAAMAGDD-------LDNIKQKLEAANKAVSKIGQHMQGGGGDTKLRKLN------ 661
Os09g31486 -----------------IK--SSGGLSESDIEKMVREAELHSQKDQERKSLIDLKNSADTTIYSIEKSVS---EYKDKVPAEVTNEIQSAVSDLRAAMAEDD-------LEKIKQKLEAANKAVSKIGEHMQQGGGGGSGGGSS------ 662
AT5G09590 -----------------IR--SSGGLSEDDIQKMVREAELHAQKDKERKELIDTKNTADTTIYSIEKSLG---EYREKIPSEIAKEIEDAVADLRSASSGDD-------LNEIKAKIEAANKAVSKIGEHMSGGSGGGSAPGGG------ 664
AT4G37910 -----------------IR--SSGGLSDDEINRMVKEAELNAQKDQEKKQLIDLRNSADTTIYSVEKSLS---EYREKIPAEIASEIETAVSDLRTAMAGED-------VEDIKAKVEAANKAVSKIGEHMSKGSGSSGSDGSSG----- 660
Os02g53420 -----------------IR--SSGGLSEAEIQKMVQEAELHSQKDQERKALIDIRNNADTTIYSVEKSLG---EYRDKIPAEVATEIETAIADLRSVMTSDD-------IEKIKANIEAANKAVSKIGQHMSGGGGGAGGS--ET----- 659
Os03g02260 -----------------IR--SSGGLSEAEIQKMVHEAELHSQKDQERKALIDIRNTADTTIYSIEKSLG---EYRDKIPAEVASEIETAIADLRNEMASDD-------IEKIKSKIEAANKAVSKIGQHMSGGGSGGSQ---------- 656
Bd3g57450 -----------------IR--SSGGLSESEIEKMVREAELHSQKDQERKALIDIRNTADTTIYSIEKSLG---EYREKIPAEIATEIETAVADLRSEMASDD-------IEKIKNKMEAANKAVSKIGQHMSGGASGGGAAGGPQ----- 661
Bd1g69700 -------------EVRMPTLDDGHGWCGQALAKMYGGNLDLAVLPKKLQP---------------------------------------------------------------------------------------------------- 578
Os03g11910 -------------EVRMPTLDDGHGWCGQALAKMYGSTLDLATIPKKLHP---------------------------------------------------------------------------------------------------- 578
AT2G32120 -------------EVRMPTVDDGHGWCAQALNVKYGATLDLITLQRKM------------------------------------------------------------------------------------------------------ 563
Bd3g53100 EKVLKKRTFRVPLKVVEKTAGAGSILSKELYSEAKNRLEVLDKKDAERRRTAELKNNLESYIYSMKEKLEENTDLLAVSTEQERESFAEKLSEVQDWLYMDE----EAQANEFQERLDQLKAIGDPILFRLSELKARPAACGSA------ 747
Os02g48110 EKVLKKRTFRVPLKVVEKMAGAGSILSKELYSEAKTRLEALDKKDAERRRTAELKNNLESYIYSMKEKLEENTEILTVSTEQERESFAEKLNEVQDWLYMDG---EDAQANEFKERLDQLKAIGDPILFRLSELKARPTACENA------ 756
AT4G16660 EKKLKKRTFRIPLKVVEKTVGPGAPFSKESLAEAKIKLEALDKKDRERRRTAELKNNLESYIYATKEKLE-TPEFEKISTQEERKAFVEKLDEVQDWLYMDG---EDANATEFEKRLDSLKAIGSPISFRSEELTARPVAIEYA------ 748
Bd1g32770 KSRNERPIQRQDLQIIGSIYGA---MSKQELLEAQEQEYQLAYQDKLMERTKERKNALESYVYDIRNKLSE--RYRSFATDSEREQISVNLQQTEEWLYEEG---DDETEEVYSSKLEELKKLVDPIENRCKDDEVRTQTTREL------ 652
Os06g46600 QVDKSRSERLIQLPIVQSIYGA---MSNQELLEAQEQESQLAYQDKLMERTKERKNALESYVYDTRNKLSE--RYRSFATDSEREEISLSLQQTEDWLYEEG---DDETEAVYNSKLEELKRLVDPIENRCKDEEVRGQATRDL------ 658
AT1G11660 IGNEPKAIKRMEIPVVANVSGA---LTKDELSEAKQRENSLVEQDLKMESTKDKKNALESFVYEMRDKMLN--TYRNTATESERECIARNLQETEEWLYEDG---DDESENAYIEKLNDVKKLIDPIENRFKDGEERVQASKDL------ 666
AT1G79920 AKAPKKKVKKTNVPLSELVYGA---LKTVEVEKAVEKEFEMALQDRVMEETKDRKNAVESYVYDMRNKLSD--KYQEYITDSEREAFLANLQEVEDWLYEDG---EDETKGVYVAKLEELKKVGDPVEVRYKESLERGSVIDQL------ 707
AT1G79930 AEAPKKKVKKTNVPLSELVYGA---LKTVEVEKAVEKEFEMALQDRVMEETKDRKNAVESYVYDMRNKLSD--KYQEYITDSEREAFLANLQEVEDWLYEDG---EDETKGVYVAKLEELKKVGDPVEVRYKESLERGSVIDQL------ 707
Os01g08560 VEPSKKKVKKTNVPVAELVYGA---LGTTELQKAVEKEYEMALQDRVMEETKDKKNAVESYVYDMRNKLYD--KYNDFVTAEDKEAFIAKLQEVEDWLYEDG---EDETKGVYVAKLEELKKVGGPIEARYKEWMDRGPSIDQL------ 702
Os05g08840 VEPLIKNVKKIDVPVSGLVYGA---LGSEELVKASENEYEMALQDRVMEETKEKKNAVEAYVYDMRNKLYD--KYNDFVMSEYKEGFIAKLQEVEDWLYEDG---EDETKGVYIAKLEELKKVGDPIEIRYKEWAERSSSINQL------ 701
    ruler .......760.......770.......780.......790.......800.......810.......820.......830.......840.......850.......860.......870.......880.......890.......900
```

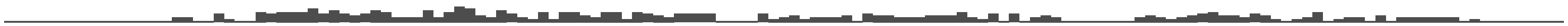

# CLUSTAL X (1.83) MULTIPLE SEQUENCE ALIGNMENT

**File: Z:Hsp reeA1.pep.ps** **Date: Tue Dec 18 15:07:21 2012**



```
AT5G28540 ---------------EEEDESHDEL----------------------------------------------------------------------------------------------------------------------------- 669
AT5G42020 ---------------EEEDESHDEL----------------------------------------------------------------------------------------------------------------------------- 668
Bd3g01480 ---------------D--DEEHDEL----------------------------------------------------------------------------------------------------------------------------- 665
Os02g02410 ---------------V--DDEHDEL----------------------------------------------------------------------------------------------------------------------------- 665
AT1G09080 ---------------E--------L----------------------------------------------------------------------------------------------------------------------------- 675
Os03g50250 ---------------NEL------------------------------------------------------------------------------------------------------------------------------------ 669
Bd2g06050 ------------------EDDHDEL----------------------------------------------------------------------------------------------------------------------------- 671
Os05g35400 ---------------DD-EDDHDEL----------------------------------------------------------------------------------------------------------------------------- 687
Os08g09770 ---------------DD-EDDHDEL----------------------------------------------------------------------------------------------------------------------------- 676
Os05g30480 ---------------GG-DDDHDEL----------------------------------------------------------------------------------------------------------------------------- 669
Os05g51360 ------------------------------------------------------------------------------------------------------------------------------------------------------ 437
Os06g10990 ------------------------------------------------------------------------------------------------------------------------------------------------------ 470
Bd4g43170 ------------------------------------------------------------------------------------------------------------------------------------------------------ 454
Os12g05760 ------------------------------------------------------------------------------------------------------------------------------------------------------ 461
Bd4g39820 ------------------------------------------------------------------------------------------------------------------------------------------------------ 438
Bd4g39850 ------------------------------------------------------------------------------------------------------------------------------------------------------ 438
AT4G24280 ASSGDSSSSKGGDGDDVIDADFTDSQ---------------------------------------------------------------------------------------------------------------------------- 718
AT5G49910 SSDTSSSAKGGDNGGDVIDADFTDSN---------------------------------------------------------------------------------------------------------------------------- 718
Bd2g30560 DS--G-PSEKPGDDGDVIDADFTDSK---------------------------------------------------------------------------------------------------------------------------- 684
Os05g23740 GSAGP-SEKPGGEEGDVIDADFTDSQ---------------------------------------------------------------------------------------------------------------------------- 689
Bd4g39470 PTPGAGPTGSSGNDGDVIDADFTDSN---------------------------------------------------------------------------------------------------------------------------- 688
Os12g14070 AGPT-SSGGKGPNDGDVIDADFTDSN---------------------------------------------------------------------------------------------------------------------------- 698
Bd4g33880 ---------TRI--PRKLRCRFN--LRQAPTFFLCVPSVGS------------------------------------------------------------------------------------------------------------- 689
Os09g31486 ---------SSSGGDQTPEAEYQDAAKEAKM----------------------------------------------------------------------------------------------------------------------- 684
AT5G09590 ---------SEGGSDQAPEAEYEEVKK--------------------------------------------------------------------------------------------------------------------------- 682
AT4G37910 -------EGT-SGTEQTPEAEFEEASGSRK------------------------------------------------------------------------------------------------------------------------ 682
Os02g53420 -------GGSQGGGEQAPEAEYEEVKK--------------------------------------------------------------------------------------------------------------------------- 679
Os03g02260 -------AGSQGGGDQAPEAEYEEVKK--------------------------------------------------------------------------------------------------------------------------- 676
Bd3g57450 -------EGSQGGGDQAPEAEYEEVKK--------------------------------------------------------------------------------------------------------------------------- 681
Bd1g69700 ------------------------------------------------------------------------------------------------------------------------------------------------------ 578
Os03g11910 ------------------------------------------------------------------------------------------------------------------------------------------------------ 578
AT2G32120 ------------------------------------------------------------------------------------------------------------------------------------------------------ 563
Bd3g53100 ---------------RLYLTELQKIVKNWETNKPWLPKKRVDEVVSEADKIRTWLDEKEALQKSTTLYSTPAFTSEEVYQKVLDLQDKVSSVNRIPKPKPKIEKKPPTEEESANK----------EKT---------ASSESTSSESEST 863
Os02g48110 ---------------RLYLAELQKIVKNWDSNKPWLPKKRVDEVVSEAEKVKTWLEEKEAIQKSTPVYSPPAFTSEEVYEKVLDLQDKVSSVNRIPKPKPKIEKKPPKEEESANK----------EKT---------DSSESESKEAEST 872
AT4G16660 ---------------RKYLTELKEIIKEWETNKTWLPKEKIDEVSKEAEKVKSWLDKNVAEQEKTSLWSKPVFTSTEVYAKVFTLQDKVTKVNKIPKPKPKIEKVTKTENTTKEE----------EQS---------KSSDEAAKEEESH 864
Bd1g32770 ---------------LKCIVDHRMAAK-------SLSAPEREAVDNECAKVEQWLREGLKLQESLPKDVDPVLWSPEIKRKEEELDMYLFFLPLRLLFTMLSSFTKMFGVSFLRRFA--------------------------------- 747
Os06g46600 ---------------LKFILDHKTAAK-------SLPTPEQEAVDSECTKAEQWLRERSQLQESLPKNVDPALWSHEIKKKEHELDMFYRNIVRYKGSPARADSSGGSDHMHTTDRD--------------------------------- 753
AT1G11660 ---------------LKTIADNRMAAE-------SLPPPRKNAVLDECHKAERWLHEKTTEQESLPKDANPELQSAEIRRKADALNATCKYIGKSNSPPAKPEHNGSYGSRKSDDMELD------------------------------- 763
AT1G79920 ---------------GYCINSYREAAVSNDPKFDHIELAEKQKVLNECVEAEAWLREKQQQQDTLPKYATPALLSADVKSKAEALDKFCRPIMTKPKPAAKAEAPQAKGGEQA---------------DEGKS----------EPEQPAS 817
AT1G79930 ---------------GYCINSYREAAMSTDPKFDHIELAEKQKVLNECVEAEAWLRGKQQQQDTLPKYATPALLSADVKSKAEALDKFCRPIMTKPKPVAKAEAPQAKGGEQA---------------DEGKS----------EPEQPAS 817
Os01g08560 ---------------AYCINSFRDAALSKDPKFDHIEMEEKQKVINQCSEAEVWLREKIQQQDALPKHANPVLLSSDLKKKAETVDRFCKPIMMKPKPAPKPQTPP-QTPPTETPAGGAQTPEQQPQGAEAAGEASE----GG--ASEST 830
Os05g08840 ---------------VHCINGFKEVALSNSQAFDHIDMSEKQKVLDECSEAEIWLIEKQQQQDALPKHADPVLLISDMKKKAEALDRSCRPIMSKPKPAPKPQTPPPPTPPTESPTTPEPQTPEQQQQSNGAGEAEEPTSEGGAQDQEPT 836
ruler .......910.......920.......930.......940.......950.......960.......970.......980.......990......1000......1010......1020......1030......1040......1050
```

```
 AT5G28540 ------------------------------   669
 AT5G42020 ------------------------------   668
 Bd3g01480 ------------------------------   665
Os02g02410 ------------------------------   665
 AT1G09080 ------------------------------   675
Os03g50250 ------------------------------   669
 Bd2g06050 ------------------------------   671
Os05g35400 ------------------------------   687
Os08g09770 ------------------------------   676
Os05g30480 ------------------------------   669
Os05g51360 ------------------------------   437
Os06g10990 ------------------------------   470
 Bd4g43170 ------------------------------   454
Os12g05760 ------------------------------   461
 Bd4g39820 ------------------------------   438
 Bd4g39850 ------------------------------   438
 AT4G24280 ------------------------------   718
 AT5G49910 ------------------------------   718
 Bd2g30560 ------------------------------   684
Os05g23740 ------------------------------   689
 Bd4g39470 ------------------------------   688
Os12g14070 ------------------------------   698
 Bd4g33880 ------------------------------   689
Os09g31486 ------------------------------   684
 AT5G09590 ------------------------------   682
 AT4G37910 ------------------------------   682
Os02g53420 ------------------------------   679
Os03g02260 ------------------------------   676
 Bd3g57450 ------------------------------   681
 Bd1g69700 ------------------------------   578
Os03g11910 ------------------------------   578
 AT2G32120 ------------------------------   563
 Bd3g53100 DTSSESDAPE-------KTDDSEPEAHDEL   886
Os02g48110 ETSSESAAPEESQSEPQKTDDLEPEAHDEL   902
 AT4G16660 DEL---------------------------   867
 Bd1g32770 ------------------------------   747
Os06g46600 ------------------------------   753
 AT1G11660 ------------------------------   763
 AT1G79920 AEAMETENPAEGST----------------   831
 AT1G79930 AEPMETENPAEGST----------------   831
Os01g08560 GEQMETDKPEGTEAA---------------   845
Os05g08840 AEQMDTDKPDG-------------------   847
     ruler ......1060......1070......1080
```
