# Supplementary material for: RNA-Seq Transcriptome Analysis and Evolution of OsEBS, a Gene Involved in Enhanced Spikelet Number per Panicle in Rice
Source: Int J Mol Sci. 2023 Jun 18;24(12):10303. doi: 10.3390/ijms241210303 (PMC10299296; doi:10.3390/ijms241210303)
Supplement: Supplementary file 1 [file ijms-24-10303-s001.zip › Figure S4.pptx]

## Slide 1
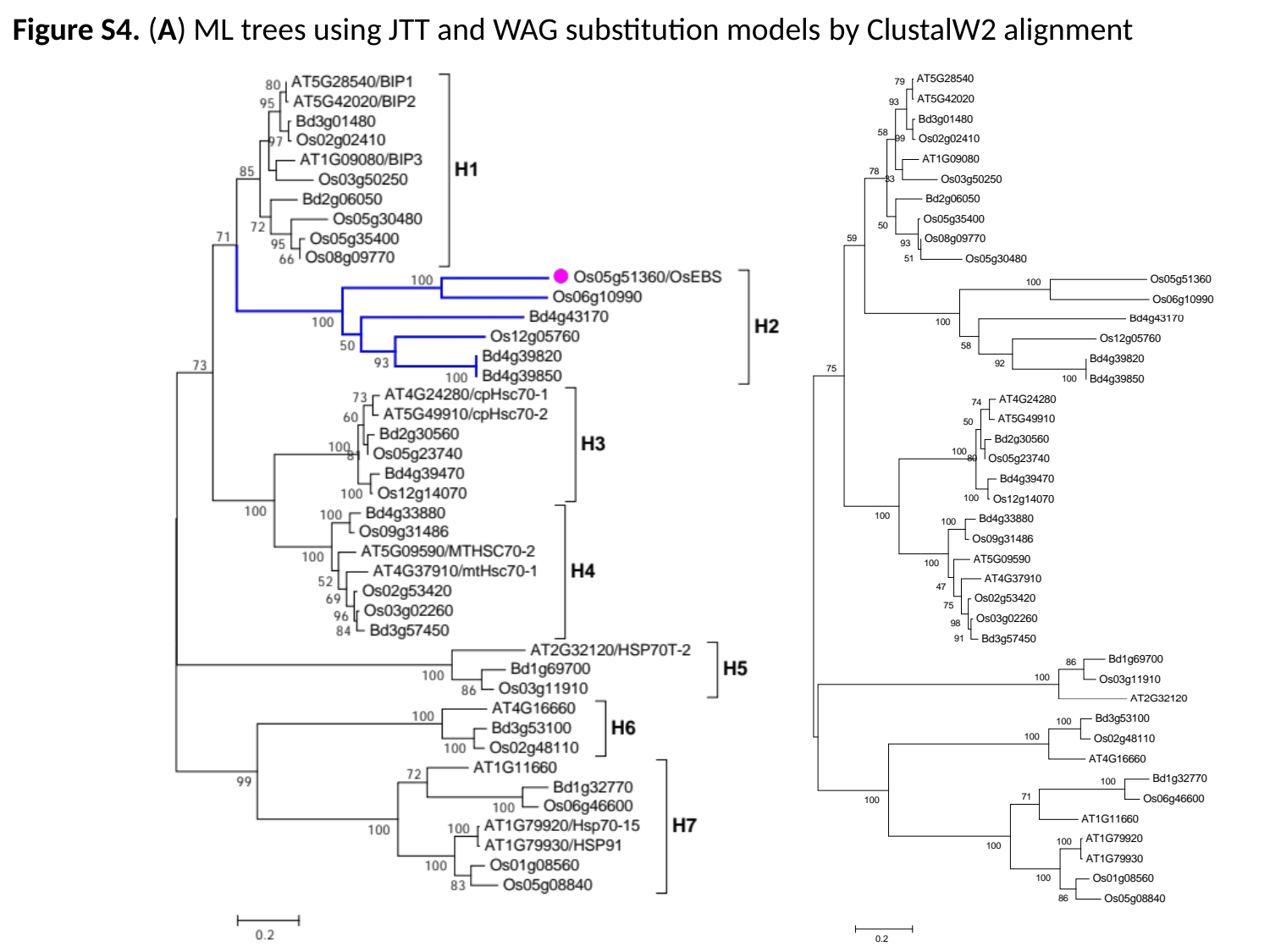

Figure S4. (A) ML trees using JTT and WAG substitution models by ClustalW2 alignment

## Slide 2
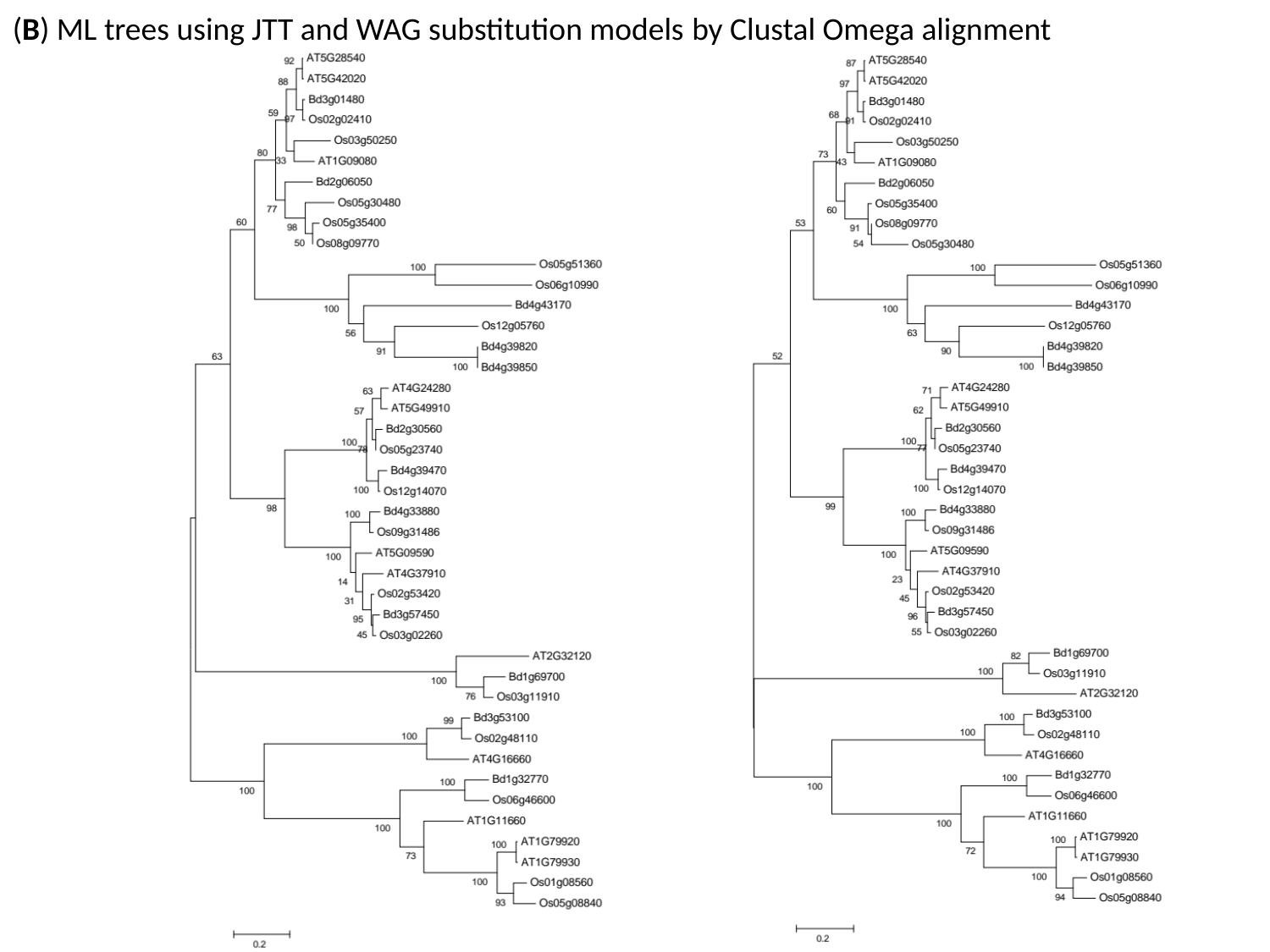

(B) ML trees using JTT and WAG substitution models by Clustal Omega alignment

## Slide 3
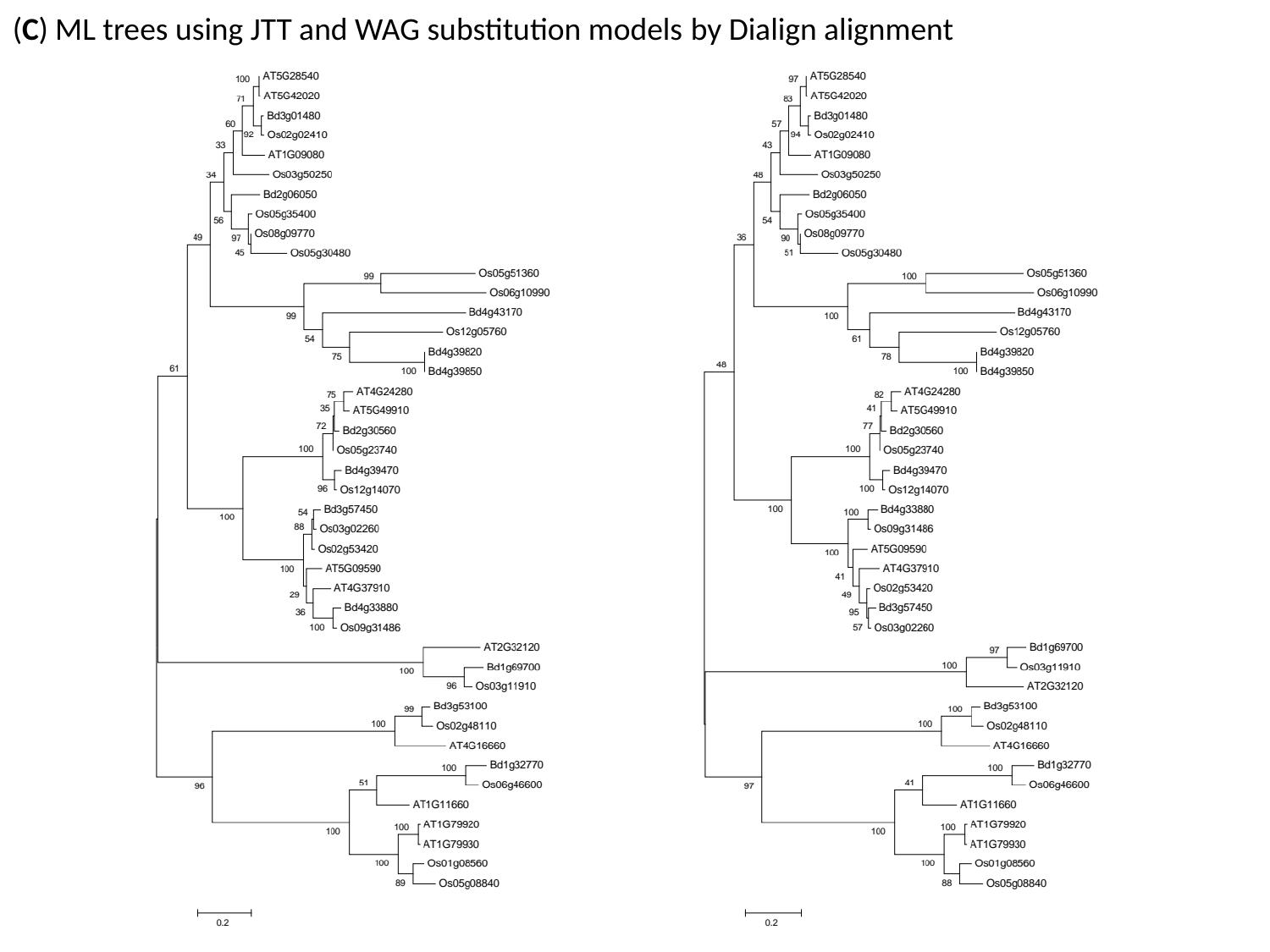

(C) ML trees using JTT and WAG substitution models by Dialign alignment

## Slide 4
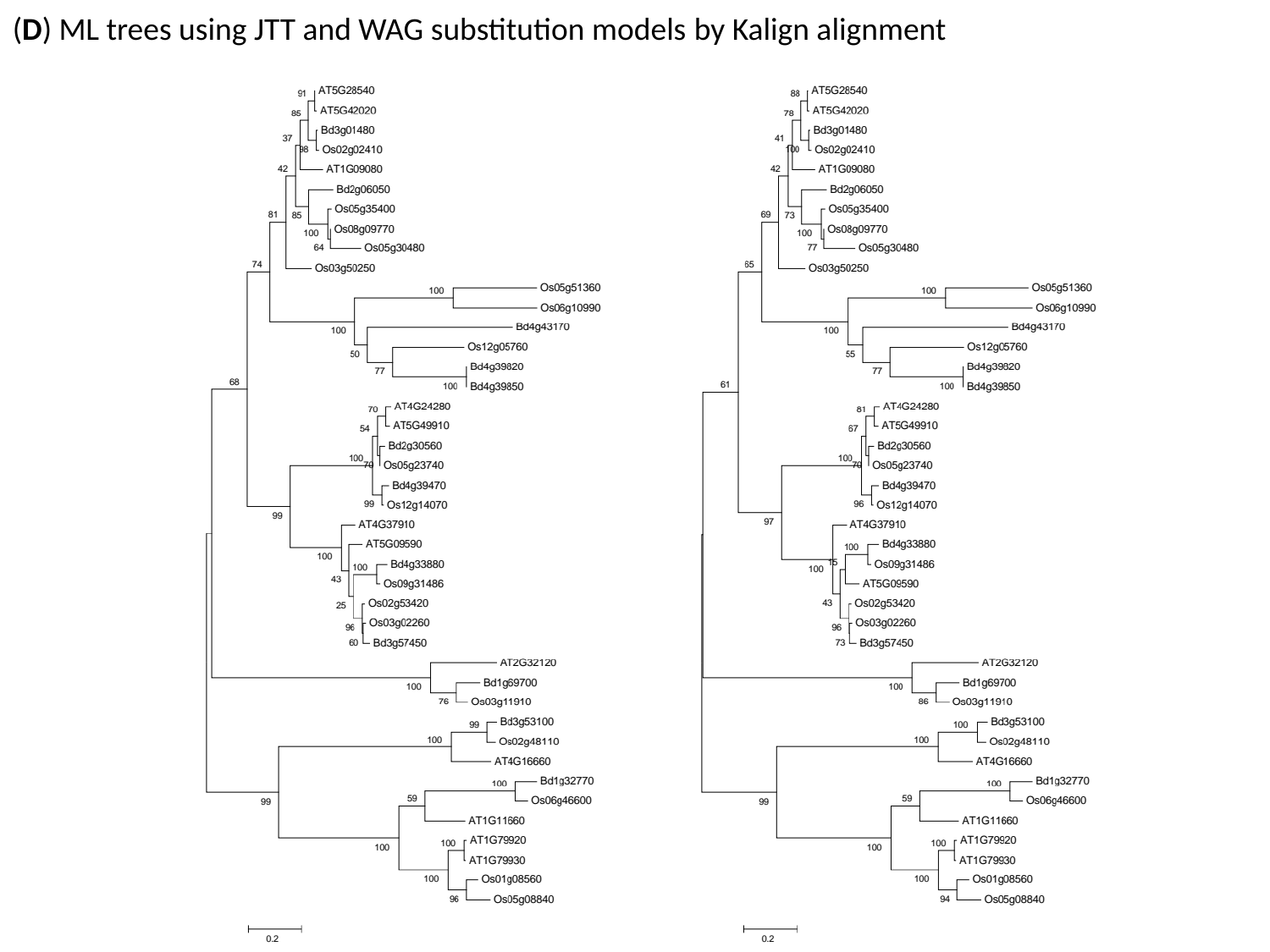

(D) ML trees using JTT and WAG substitution models by Kalign alignment

## Slide 5
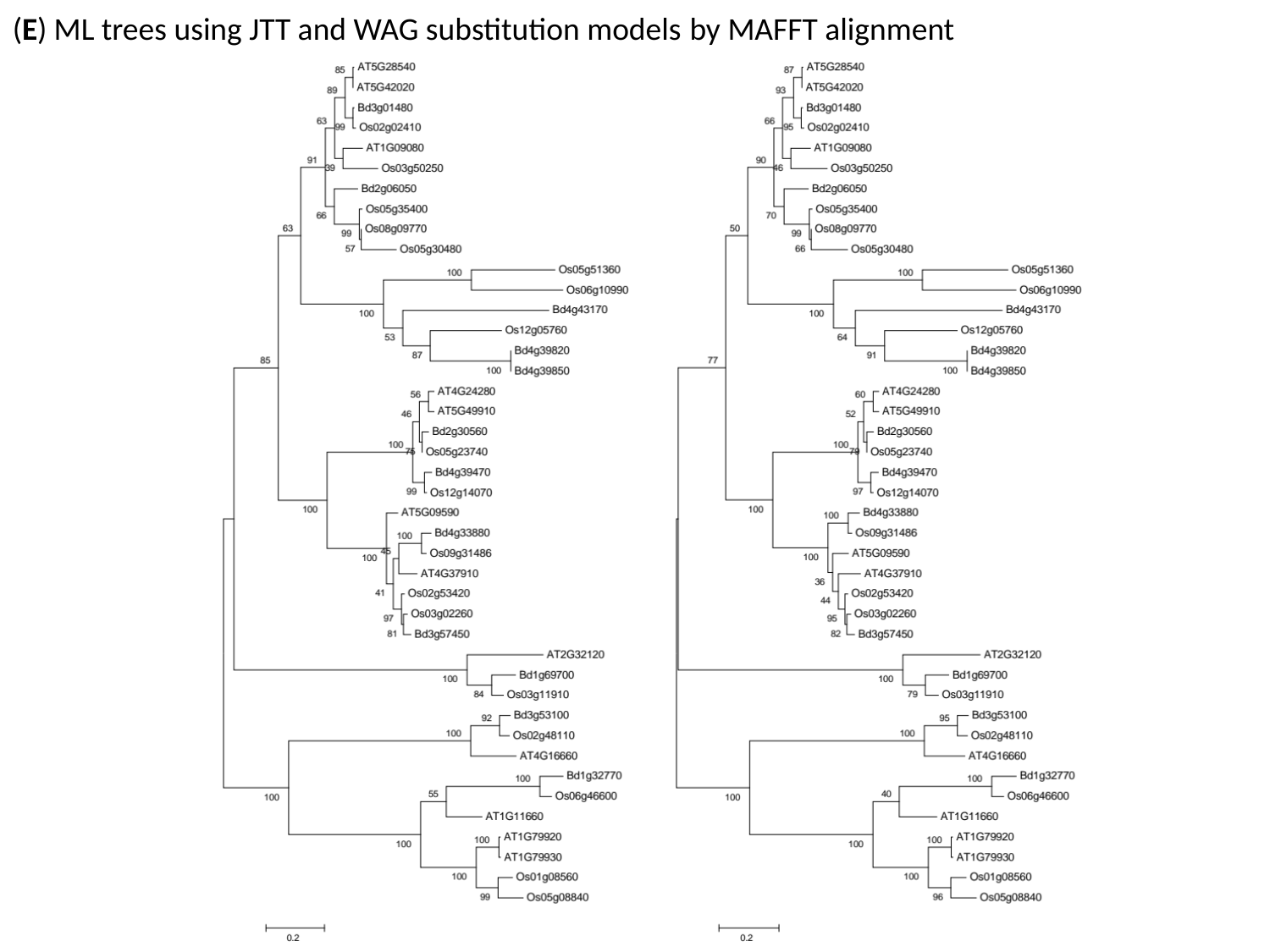

(E) ML trees using JTT and WAG substitution models by MAFFT alignment

## Slide 6
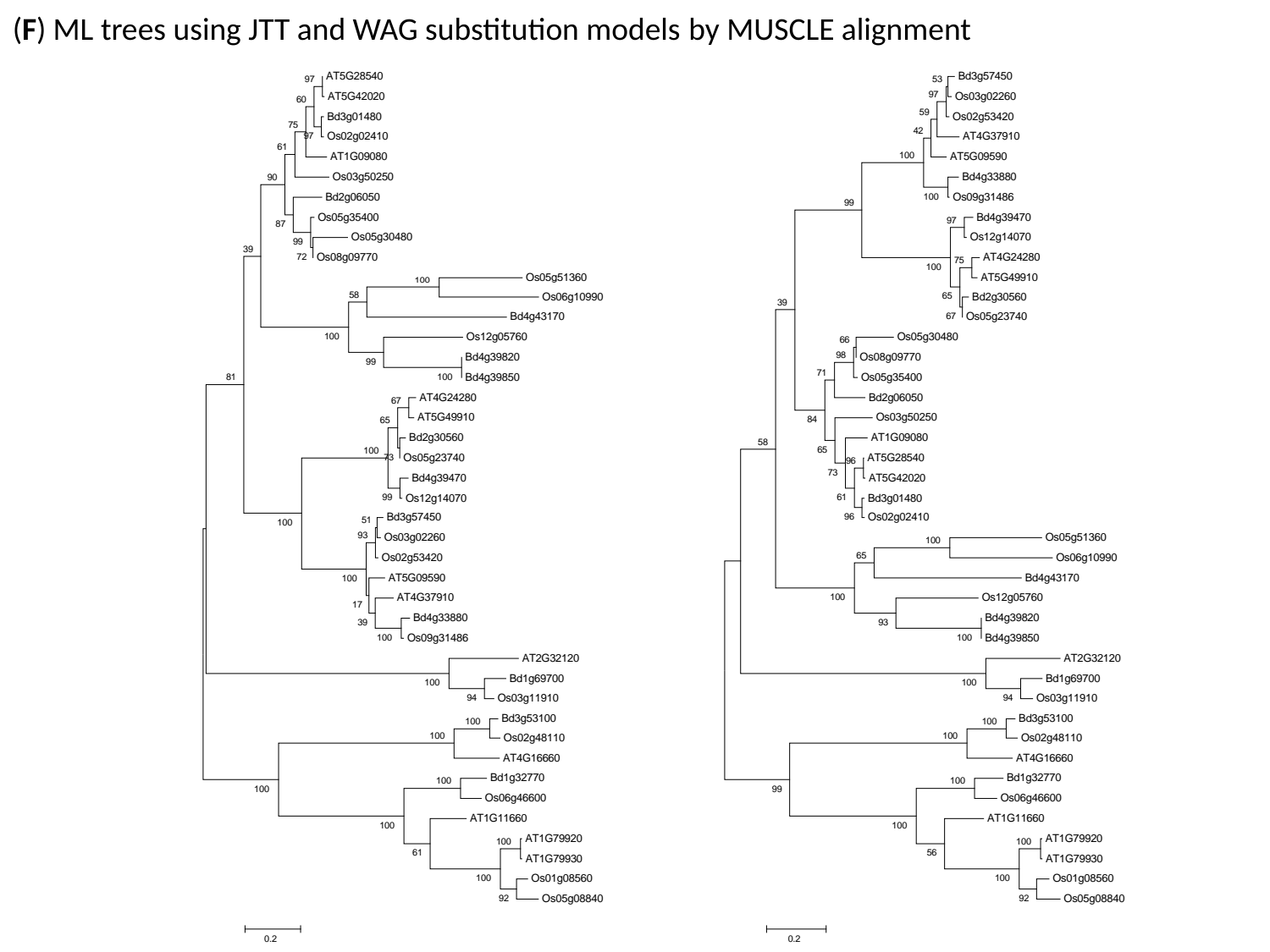

(F) ML trees using JTT and WAG substitution models by MUSCLE alignment

## Slide 7
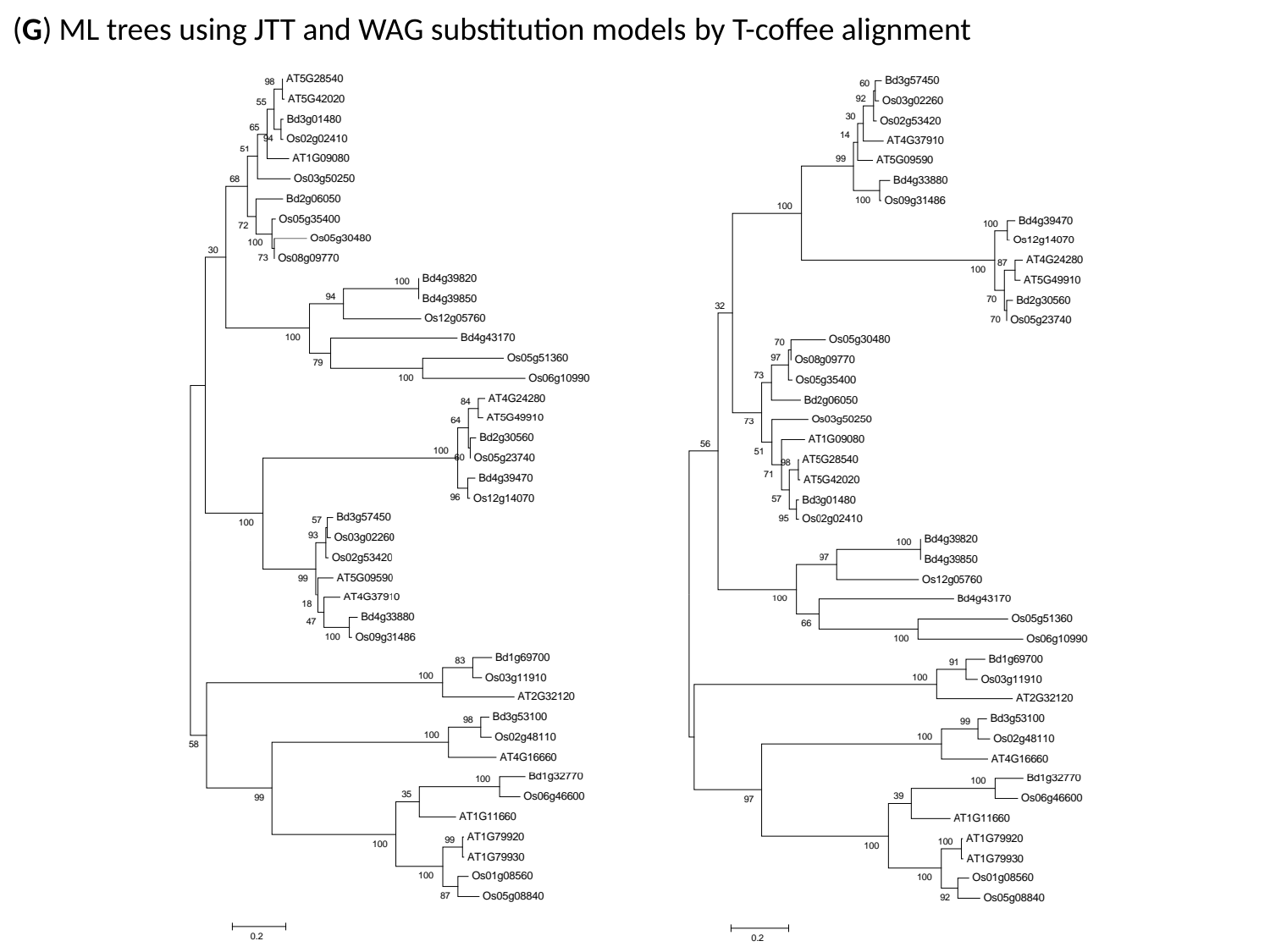

(G) ML trees using JTT and WAG substitution models by T-coffee alignment
