# Supplementary material for: RNA-Seq Transcriptome Analysis and Evolution of OsEBS, a Gene Involved in Enhanced Spikelet Number per Panicle in Rice
Source: Int J Mol Sci. 2023 Jun 18;24(12):10303. doi: 10.3390/ijms241210303 (PMC10299296; doi:10.3390/ijms241210303)
Supplement: Supplementary file 1 [file ijms-24-10303-s001.zip › Table S1-S8.pdf]

Table S1. Statistical table for mapping rate of samples.

Library	B1021	B1022	B1023	G21	G22	G23
Total Reads	40,652,450	45,029,452	46,814,038	39,480,362	44,404,198	38,709,036
Mapped Reads	35,415,808	38,796,427	40,559,649	33,835,636	38,465,065	33,483,682
Mapping Rate	0.8712	0.8616	0.8664	0.8570	0.8662	0.8650
UnMapped Reads	5,236,642	6,233,025	6,254,389	5,644,726	5,939,133	5,225,354
MultiMap Reads	939,802	1,026,897	1,043,893	795,564	982,602	879,232
MultiMap Rate	0.0231	0.0228	0.0223	0.0202	0.0221	0.0227

Table S2. Analysis of DEGs between different materials.

Name	B1021_G21	B1021_G22	B1022_G21	B1022_G22	B1022_G23	B1023_G21	B1023_G22	B1023_G23	B_G
Up	1,507	1,549	1,484	1,615	1,602	1,451	1,637	1,721	1,204
Down	5,435	5,201	5,062	4,921	4,367	4,775	4,614	4,149	4,165
Total	6,942	6,750	6,546	6,536	5,969	6,226	6,251	5,870	5,369

Table S3. DEGs related to rice auxin regulation in rice.

	Gene	Fold Change	q Value	Putative Function
Up regulated DEGs	OsZS_11G0101800	1.68	0.0001	F-box/LRR-repeat protein 13
	OsZS_11G0039500	1.66	0.0000	Probable auxin efflux carrier component 1b
	OsZS_06G0600900	1.53	0.0000	Molybdenum cofactor sulfurase
	OsZS_12G0038200	1.46	0.0000	Probable auxin efflux carrier component 1d
	OsZS_02G0541900	1.46	0.0398	VAN3-binding protein
	OsZS_05G0060500	1.45	0.0000	ABC transporter B family member 11
	OsZS_11G0101900	1.35	0.0000	F-box/LRR-repeat protein At4g14096
	OsZS_04G0788300	1.22	0.0000	Auxin response factor 11
	OsZS_06G0098700	1.17	0.0000	BTB/POZ domain-containing protein NPY1
	OsZS_05G0580100	1.16	0.0000	Auxin response factor 15
	OsZS_04G0788200	1.11	0.0027	Auxin response factor 11
	OsZS_04G0769600	1.02	0.0000	ABC transporter B family member 19
	OsZS_05G0540900	1.01	0.0000	Auxin response factor 14
	OsZS_10G0056400	1.00	0.0000	Auxin transporter-like protein 3
	–			
Down regulated DEGs	OsZS_01G0558800	**-5.06**	**0.0000**	**ABC transporter B family member 4**
	OsZS_05G0165300	-4.65	0.0000	CASP-like protein BLE3
	OsZS_03G0652400	-4.29	0.0000	Transcription factor MYB44
	OsZS_12G0491700	-3.90	0.0000	NAC domain-containing protein 21/22
	OsZS_05G0603900	-3.62	0.0000	Bidirectional sugar transporter SWEET5
	OsZS_11G0101400	-3.60	0.0000	Transcription factor MYB44
	OsZS_12G0037400	-3.49	0.0000	Protein ZINC INDUCED FACILITATOR-LIKE 1
	OsZS_02G0022000	-3.40	0.0000	Protein WALLS ARE THIN 1
	OsZS_02G0071100	-3.33	0.0000	NAC domain-containing protein 21/22
	OsZS_02G0112100	-3.30	0.0000	Subtilisin-like protease SBT5.3
	OsZS_06G0580300	-2.89	0.0000	AP2-like ethylene-responsive transcription factor AIL5
	OsZS_09G0018600	-2.81	0.0000	Transcription factor MYB44
	OsZS_04G0586500	-2.60	0.0000	2-oxoglutarate-dependent dioxygenase DAO
	OsZS_06G0612500	-2.57	0.0000	NAC domain-containing protein 21/22
	OsZS_02G0702000	-2.48	0.0000	NAC domain-containing protein 21/22
	OsZS_03G0012400	-2.42	0.0003	Cortical cell-delineating protein
	OsZS_12G0491200	-2.41	0.0001	Transcription factor PIF4
	OsZS_12G0037500	-2.33	0.0004	Protein ZINC INDUCED FACILITATOR-LIKE 1
	OsZS_03G0734600	-2.31	0.0005	Probable aldehyde oxidase 2
	OsZS_03G0734400	-2.25	0.0000	Probable aldehyde oxidase 2
	OsZS_02G0463000	-2.18	0.0011	2-oxoglutarate-dependent dioxygenase DAO
	OsZS_04G0052300	-2.13	0.0000	Probable indole-3-pyruvate monooxygenase **YUCCA3**
	OsZS_10G0401000	-2.04	0.0000	Auxin-responsive protein SAUR32
	OsZS_01G0195900	-1.99	0.0000	Auxin-responsive protein **IAA**4
	OsZS_11G0050900	-1.97	0.0000	Serine/threonine-protein kinase D6PKL2
	OsZS_03G0012200	-1.78	0.0002	Protein NRT1/ PTR FAMILY 6.3
	OsZS_07G0096400	-1.78	0.0099	Putative auxin response factor 20
	OsZS_06G0563500	-1.75	0.0000	Transcription factor MYB44
	OsZS_04G0779200	-1.74	0.0089	Auxin-responsive protein IAA33

OsZS_05G0063000	-1.73	0.0136	Pyrophosphate-energized vacuolar membrane proton pump 1
OsZS_11G0039000	-1.71	0.0132	Protein ZINC INDUCED FACILITATOR-LIKE 1
OsZS_02G0100900	-1.71	0.0000	Transcription factor MYB44
OsZS_04G0647200	-1.63	0.0196	Auxin-responsive protein SAUR36
OsZS_06G0631400	-1.59	0.0000	Xyloglucan endotransglucosylase/hydrolase protein 22
OsZS_06G0047000	-1.57	0.0000	Auxin-responsive protein SAUR50
OsZS_05G0051600	-1.52	0.0021	Probable indole-3-acetic acid-amido synthetase GH3.6
OsZS_01G0643700	-1.52	0.0000	NAD(P)H dehydrogenase (quinone) FQR1
OsZS_09G0415200	-1.51	0.0000	Protein PIN-LIKES 7
OsZS_01G0566600	-1.51	0.0060	Cullin-1
OsZS_01G0791800	-1.50	0.0127	Probable glutathione S-transferase
OsZS_03G0542100	-1.46	0.0305	ARF guanine-nucleotide exchange factor GNOM
OsZS_08G0637000	-1.41	0.0000	Auxin-responsive protein SAUR36
OsZS_01G0198600	-1.41	0.0000	ABC transporter B family member 21
OsZS_02G0609900	-1.38	0.0000	Auxin-responsive protein IAA8
OsZS_01G0644100	-1.37	0.0000	Probable indole-3-acetic acid-amido synthetase GH3.1
OsZS_09G0304900	-1.35	0.0292	Auxin-responsive protein SAUR50
OsZS_02G0281400	-1.32	0.0000	Auxin-responsive protein SAUR50
OsZS_02G0657200	-1.27	0.0061	Auxin-responsive protein SAUR32
OsZS_08G0010700	-1.26	0.0070	NAC domain-containing protein 30
OsZS_03G0171700	-1.26	0.0000	Trifunctional UDP-glucose 4,6-dehydratase/UDP-4-keto-6-deoxy-D-glucose 3,5-epimerase/UDP-4-keto-L-rhamnose-reductase RHM1
OsZS_08G0655500	-1.25	0.0030	ABC transporter B family member 1
OsZS_03G0073600	-1.23	0.0003	Protein BIG GRAIN 1
OsZS_11G0065400	-1.21	0.0000	Putative auxin transporter-like protein 4
OsZS_04G0632900	-1.15	0.0000	Auxin response factor 10
OsZS_03G0563800	-1.14	0.0000	Type IV inositol polyphosphate 5-phosphatase 11
OsZS_02G0351400	-1.14	0.0000	Flavonoid 3'-monooxygenase
OsZS_01G0183100	-1.11	0.0482	Probable indole-3-pyruvate monooxygenase YUCCA10
OsZS_03G0567700	-1.11	0.0050	Auxin-responsive protein IAA12
OsZS_11G0549000	-1.07	0.0000	Glycine-rich domain-containing protein 2
OsZS_05G0046000	-1.06	0.0000	ABC transporter B family member 4
OsZS_07G0491000	-1.06	0.0020	Probable indole-3-acetic acid-amido synthetase GH3.8
OsZS_05G0549100	-1.05	0.0000	Auxin-responsive protein IAA18
OsZS_06G0521900	-1.02	0.0283	Auxin-responsive protein IAA23

Table S4. Enriched KEGG metabolic pathways (Top 30).

Number	Name	q Value	Up_ Count	Down_ Count	Down DEGs related to auxin regulation
1	Phenylpropanoid biosynthesis	0.0000	2	67	
2	Cutin, suberine and wax biosynthesis	0.0003	1	22	
3	Cyanoamino acid metabolism	0.0027	0	13	
4	Photosynthesis - antenna proteins	0.0113	0	7	
5	Glutathione metabolism	0.0136	1	25	
6	alpha-Linolenic acid metabolism	0.0261	0	14	
7	Valine, leucine and isoleucine degradation	0.0301	1	14	
8	Amino sugar and nucleotide sugar metabolism	0.0333	1	33	OsZS_03G0171700
9	Pentose and glucuronate interconversions	0.0333	0	14	
10	Photosynthesis	0.0396	0	14	
11	Flavonoid biosynthesis	0.0499	0	9	OsZS_02G0351400
12	Tropane, piperidine and pyridine alkaloid biosynthesis	0.0523	0	7	
13	Fatty acid elongation	0.0780	1	13	
14	MAPK signaling pathway - plant	0.0780	5	28	
15	Mannose type O-glycan biosynthesis	0.1243	0	4	
16	Phenylalanine metabolism	0.1243	1	8	
17	Ascorbate and aldarate metabolism	0.1273	0	11	
18	Starch and sucrose metabolism	0.1273	5	24	
19	beta-Alanine metabolism	0.1273	0	10	
20	Anthocyanin biosynthesis	0.2509	0	7	
21	Stilbenoid, diarylheptanoid and gingerol biosynthesis	0.2571	0	6	
22	Tyrosine metabolism	0.3849	0	6	
23	Homologous recombination	0.3849	18	1	
24	Fatty acid degradation	0.3849	0	10	
25	Benzoxazinoid biosynthesis	0.3994	0	5	
26	ABC transporters	0.3994	3	5	OsZS_01G0558800; OsZS_08G0655500;OsZS_05G0046000
27	Plant-pathogen interaction	0.3994	7	50	
28	Pyrimidine metabolism	0.4010	14	10	
29	Carbon fixation in photosynthetic organisms	0.4129	0	13	
30	Phenylalanine, tyrosine and tryptophan biosynthesis	0.4686	1	10	

Table S5. Distribution of *OsEBS* full-length and deletion allele types in indica, japonica and wild rice.

Accessions	Variety	Species	*OsEBS* gene type
1	TR24	indica	Full-length
2	Baxihandao	indica	Full-length
3	Ezao18	indica	Full-length
4	Haixianxiang5hao	indica	Full-length
5	Huhan15hao	indica	Full-length
6	Jiayu293	indica	Full-length
7	Jinzao47	indica	Full-length
8	Taiyoujingzhan	indica	Full-length
9	TianfengB	indica	Full-length
10	Wuyouyuehesimiao	indica	Full-length
11	Zhonghui8015	indica	Full-length
12	Zhongjian100	indica	Full-length
13	Zhongzao39	indica	Full-length
14	ZhongzheB	indica	Full-length
15	Arias	indica	Truncated
16	Dhala	indica	Truncated
17	Dholi Boro	indica	Truncated
18	DV1	indica	Truncated
19	DV85	indica	Truncated
20	Fan31	indica	Truncated
21	Fan32	indica	Truncated
22	Fan38	indica	Truncated
23	Fan39	indica	Truncated
24	Francis	indica	Truncated
25	IR64	indica	Truncated
26	Jupiter	indica	Truncated
27	KU115	indica	Truncated
28	Mansaku	indica	Truncated
29	N22	indica	Truncated
30	Padi Boenor	indica	Truncated
31	shada Boro	indica	Truncated
32	Shuo1	indica	Truncated
33	**T1**	indica	Truncated
34	Taducan	indica	Truncated
35	weus/Rordo	indica	Truncated
36	Baguixiang	indica	Truncated
37	Beijingjiangminuo	indica	Truncated
38	Fuhui838	indica	Truncated
39	Guangtouhulu	indica	Truncated
40	Hongnuo822	indica	Truncated
41	Jiuyuehuang	indica	Truncated
42	Keyuanyou610	indica	Truncated
43	Liushizao	indica	Truncated
44	Lucaihao	indica	Truncated
45	Neixiang5B	indica	Truncated
46	Qiuqianbai	indica	Truncated

47	Sanbaili	indica	Truncated
48	Shundetangmu	indica	Truncated
49	Taidongludao	indica	Truncated
50	XieB	indica	Truncated
51	Yudao1hao	indica	Truncated
52	Zaoyouzhan	indica	Truncated
53	Zhong9B	indica	Truncated
54	Zhongzao22	indica	Truncated
55	Zhouandao	indica	Truncated
56	489S	japonica	Full-length
57	77B	japonica	Full-length
58	8527B	japonica	Full-length
59	9520B	japonica	Full-length
60	98101B	japonica	Full-length
61	AB21	japonica	Full-length
62	Fan1	japonica	Full-length
63	Fan10	japonica	Full-length
64	Fan11	japonica	Full-length
65	Fan12	japonica	Full-length
66	Fan13	japonica	Full-length
67	Fan14	japonica	Full-length
68	Fan16	japonica	Full-length
69	Fan17	japonica	Full-length
70	Fan21	japonica	Full-length
71	Fan24	japonica	Full-length
72	Fan25	japonica	Full-length
73	Fan26	japonica	Full-length
74	Fan29	japonica	Full-length
75	Fan3	japonica	Full-length
76	Fan33	japonica	Full-length
77	Fan34	japonica	Full-length
78	Fan35	japonica	Full-length
79	Fan36	japonica	Full-length
80	Fan37	japonica	Full-length
81	Fan4	japonica	Full-length
82	Fan40	japonica	Full-length
83	Fan41	japonica	Full-length
84	Fan42	japonica	Full-length
85	Fan43	japonica	Full-length
86	Fan6	japonica	Full-length
87	Fan7	japonica	Full-length
88	Fan9	japonica	Full-length
89	Ru0801030	japonica	Full-length
90	Ru1001102	japonica	Full-length
91	T65	japonica	Full-length
92	Tainan	japonica	Full-length
93	W6599	japonica	Full-length
94	W96B	japonica	Full-length
95	WDR129	japonica	Full-length

96	Baonong34	japonica	Full-length
97	DanK9B	japonica	Full-length
98	Dongbeizaodao	japonica	Full-length
99	Feidongtangdao	japonica	Full-length
100	FengJin	japonica	Full-length
101	Ganningjing1hao	japonica	Full-length
102	HanfengB	japonica	Full-length
103	Huhan106	japonica	Full-length
104	Huhan3hao	japonica	Full-length
105	Huruan1212	japonica	Full-length
106	Huayu1hao	japonica	Full-length
107	Jijing88	japonica	Full-length
108	Jia58	japonica	Full-length
109	Jindao106	japonica	Full-length
110	Jingyue1hao	japonica	Full-length
111	Kongyu131	japonica	Full-length
112	Lianjing4hao	japonica	Full-length
113	Liaoyan294	japonica	Full-length
114	Longdao203	japonica	Full-length
115	Longjing680	japonica	Full-length
116	Nanjing44	japonica	Full-length
117	Nanjing9108	japonica	Full-length
118	Ninghui8hao	japonica	Full-length
119	Ningjing3hao	japonica	Full-length
120	Ningjing7hao	japonica	Full-length
121	Nongkan57	japonica	Full-length
122	Pujing223	japonica	Full-length
123	Qianjingxiangnuo	japonica	Full-length
124	QingchangB	japonica	Full-length
125	Qingxiangruannuo	japonica	Full-length
126	qiuguang	japonica	Full-length
127	Qiutianxiaoding	japonica	Full-length
128	Shen21B	japonica	Full-length
129	Shennong9741	japonica	Full-length
130	Sidao16hao	japonica	Full-length
131	Song5513B	japonica	Full-length
132	Songjing22	japonica	Full-length
133	Songjing9hao	japonica	Full-length
134	Songnuo2hao	japonica	Full-length
135	Songxiangjing11855	japonica	Full-length
136	Songnian1hao	japonica	Full-length
137	Songnian3hao	japonica	Full-length
138	Tianlong104	japonica	Full-length
139	Tianlong660	japonica	Full-length
140	Wu1B	japonica	Full-length
141	Wuyujing7hao	japonica	Full-length
142	Wuyujing23	japonica	Full-length
143	Wuyujing27	japonica	Full-length
144	Xin600S	japonica	Full-length

145	Xu32075	japonica	Full-length
146	XienuoB	japonica	Full-length
147	Yangjing3491	japonica	Full-length
148	Yangjing7035	japonica	Full-length
149	Yangjingnuo2hao	japonica	Full-length
150	Yejinghui	japonica	Full-length
151	Yinguang	japonica	Full-length
152	Yinyexuanfei	japonica	Full-length
153	Yushuxiang	japonica	Full-length
154	Yunguang104	japonica	Full-length
155	Yunjingyou12hao	japonica	Full-length
156	Yunjingyou15hao	japonica	Full-length
157	Yunjingyou17hao	japonica	Full-length
158	Zao98110	japonica	Full-length
159	Zhendao88	japonica	Full-length
160	Zhenxiang24	japonica	Full-length
161	BJ1	japonica	Truncated
162	Fan2	japonica	Truncated
163	Daohuaxiang2hao	japonica	Truncated
164	Huhan7hao	japonica	Truncated
165	Ning5047	japonica	Truncated
166	Xiushui110	japonica	Truncated
167	Yangjing4308	japonica	Truncated
168	Zhongdan4hao	japonica	Truncated
169	Heimangdao	japonica	Full-length
170	Yeshengdao363	*O.rufipogon*	Truncated
171	Yeshengdao455	*O.rufipogon*	Truncated
172	Yeshengdao465	*O.rufipogon*	Truncated
173	Yeshengdao488	*O.rufipogon*	Truncated
174	Nov-93	indica	Truncated
175	187R	indica	Truncated
176	8INDESHWARI	indica	Truncated
177	ADT(R)48	indica	Truncated
178	AG7	indica	Truncated
179	AS 996-1K	indica	Truncated
180	Bo B	indica	Truncated
181	CIMELATI	indica	Truncated
182	CT 15672	indica	Truncated
183	CT 15691	indica	Truncated
184	CT 15765	indica	Truncated
185	CT 18232	indica	Truncated
186	CT 18233	indica	Truncated
187	CT 18243	indica	Truncated
188	CT 18372	indica	Truncated
189	CT 18375	indica	Truncated
190	CT 18597	indica	Truncated
191	CT 18599	indica	Truncated
192	CT 18620	indica	Truncated
193	CT 18657	indica	Truncated

194	CT 18667	indica	Truncated
195	CT 18685	indica	Truncated
196	CT 19561	indica	Truncated
197	CT I8148	indica	Truncated
198	Dalidao	indica	Truncated
199	Dular	indica	Truncated
200	Guichao 2	indica	Truncated
201	Hui9B	indica	Truncated
202	IR 01N223	indica	Truncated
203	IR 04N114	indica	Truncated
204	IR 09A228	indica	Truncated
205	IR 19661	indica	Truncated
206	IR 19746	indica	Truncated
207	IR 54751	indica	Truncated
208	IR 63896	indica	Truncated
209	IR 65482	indica	Truncated
210	IR 65483	indica	Truncated
211	IR 65620	indica	Truncated
212	IR 68450	indica	Truncated
213	IR 70175	indica	Truncated
214	IR 71033	indica	Truncated
215	IR 71146	indica	Truncated
216	IR 71701	indica	Truncated
217	IR 72158	indica	Truncated
218	IR 72870	indica	Truncated
219	IR 73546	indica	Truncated
220	IR 73707	indica	Truncated
221	IR 73718	indica	Truncated
222	IR 74284	indica	Truncated
223	IR 75870	indica	Truncated
224	IR 76479	indica	Truncated
225	IR 77298	indica	Truncated
226	IR 77674	indica	Truncated
227	IR 77736	indica	Truncated
228	IR 78091	indica	Truncated
229	IR 78101	indica	Truncated
230	IR 78545	indica	Truncated
231	IR 78875	indica	Truncated
232	IR 78877	indica	Truncated
233	IR 78914	indica	Truncated
234	IR 79482	indica	Truncated
235	IR 79525	indica	Truncated
236	IR 79532	indica	Truncated
237	IR 79584	indica	Truncated
238	IR 79637	indica	Truncated
239	IR 79913	indica	Truncated
240	IR 80312	indica	Truncated
241	IR 80314	indica	Truncated
242	IR 80315	indica	Truncated

243	IR 80404	indica	Truncated
244	IR 80655	indica	Truncated
245	IR 80692	indica	Truncated
246	IR 80694	indica	Truncated
247	IR 80707	indica	Truncated
248	IR 80922	indica	Truncated
249	IR 81336	indica	Truncated
250	IR 81423	indica	Truncated
251	IR 82098	indica	Truncated
252	IR 83222	indica	Truncated
253	IR 84233	indica	Truncated
254	IRRI 134	indica	Truncated
255	Liantangzao 3	indica	Truncated
256	lR 79253	indica	Truncated
257	Luzhan 7	indica	Truncated
258	Minghui 63	indica	Truncated
259	Nanjing 16	indica	Truncated
260	NEAMAT	indica	Truncated
261	OM 5338	indica	Truncated
262	OM 576	indica	Truncated
263	OM 6073	indica	Truncated
264	OM 6378	indica	Truncated
265	OM 7938	indica	Truncated
266	OMCS 2009	indica	Truncated
267	Peiai64	indica	Truncated
268	PR 26703	indica	Truncated
269	RADHA4	indica	Truncated
270	S 3429	indica	Truncated
271	Teqing2	indica	Truncated
272	UPR 84-21	indica	Truncated
273	XiangaiB	indica	Truncated
274	XieqingzaoB	indica	Truncated
275	YuefengB	indica	Truncated
276	IR 70213	indica	Full-length
277	IR 72768	indica	Full-length
278	IR 80098	indica	Full-length
279	IR 82127	indica	Full-length
280	IR 82228	indica	Full-length
281	IRBB	indica	Full-length
282	OM 5628	indica	Full-length
283	Yuedongdao	indica	Full-length
284	Zaozhenzhu	indica	Full-length
285	80A90YR72	indica	Full-length
286	CB06-505	japonica	Truncated
287	2428	japonica	Truncated
288	IR 83245	japonica	Full-length
289	IR 83265	japonica	Full-length
290	AnnongwanjingB	japonica	Full-length
291	C418	japonica	Full-length

292	CW23	japonica	Full-length
293	Dongjin	japonica	Full-length
294	G2 948	japonica	Full-length
295	GZ 7712	japonica	Full-length
296	Hexi 15	japonica	Full-length
297	Hwayoung	japonica	Full-length
298	IRBL	japonica	Full-length
299	ITAI48	japonica	Full-length
300	Jingjing 7	japonica	Full-length
301	JODO	japonica	Full-length
302	Kunjing 4	japonica	Full-length
303	Lijiangxintuanheigu	japonica	Full-length
304	lRAT4	japonica	Full-length
305	Niangaodao	japonica	Full-length
306	Ningjing 37	japonica	Full-length
307	nipponbare	japonica	Full-length
308	OLBYE 2	japonica	Full-length
309	ORO	japonica	Full-length
310	Shennong 265	japonica	Full-length
311	Taichung65	japonica	Full-length
312	Wuyugeng 3	japonica	Full-length
313	Xinzhunuo 4	japonica	Full-length
314	Yueguang	japonica	Full-length
315	YUNLEN	japonica	Full-length
316	Zhonghua 11	japonica	Full-length
317	0.alater wild rice	*o.rufipogon*	Truncated
318	Jinghong wild rice	*o.rufipogon*	Truncated
319	Miandian wild rice	*o.rufipogon*	Truncated
320	Taiwan wild rice	*o.rufipogon*	Truncated
321	YD2-0225	*O.rufipogon*	Truncated
322	YD2-0233	*O.rufipogon*	Truncated
323	YD2-0239	*O.rufipogon*	Truncated
324	YD2-0241	*O.rufipogon*	Truncated
325	YD2-0245	*O.rufipogon*	Truncated
326	YD2-0344	*O.rufipogon*	Truncated
327	YD2-0356	*O.rufipogon*	Truncated
328	YD2-0366	*O.rufipogon*	Truncated
329	YD2-0368	*O.rufipogon*	Truncated
330	YD2-0772	*O.rufipogon*	Truncated
331	YD2-0267	*O.rufipogon*	Truncated
332	YD2-0275	*O.rufipogon*	Truncated
333	YD2-0277	*O.rufipogon*	Truncated
334	YD2-0782	*O.rufipogon*	Truncated
335	Dongxiang wild rice	*O.rufipogon*	Full-length
336	YD2-0069	*O.rufipogon*	Full-length
337	YD2-0975	*O.rufipogon*	Full-length
338	YD2-0645	*O.rufipogon*	Full-length
339	YD2-0860	*O.rufipogon*	Full-length
340	YD4-24	*O.rufipogon*	Full-length

341	YD4-52	*O.rufipogon*	Full-length
342	YD4-65	*O.rufipogon*	Full-length
343	YD4-127	*O.rufipogon*	Full-length
344	YD4-191	*O.rufipogon*	Full-length
345	YD4-197	*O.rufipogon*	Full-length
346	YD4-133	*O.rufipogon*	Full-length
347	IRGC100189	*O.rufipogon*	Full-length
348	IRGC100208	*O.rufipogon*	Full-length
349	IRGC10211	*O.rufipogon*	Full-length
350	IRGC100588	*O.rufipogon*	Full-length
351	IRGC100599	*O.rufipogon*	Full-length
352	IRGC100904	*O.rufipogon*	Full-length
353	IRGC100920	*O.rufipogon*	Full-length
354	IRGC100923	*O.rufipogon*	Full-length
355	IRGC100926	*O.rufipogon*	Full-length
356	IRGC103822	*O.rufipogon*	Full-length
357	IRGC103850	*O.rufipogon*	Full-length
358	IRGC103884	*O.rufipogon*	Full-length
359	IRGC100195	*O.NIVARA*	Truncated
360	IRGC100196	*O.NIVARA*	Truncated
361	IRGC100897	*O.NIVARA*	Truncated
362	IRGC100898	*O.NIVARA*	Full-length

"Truncated" means that the protein in this variety was domain-loss like that in Guichao2;
"Full-lenght" means that the protein in this variety was full size like that in B102;
Accessions 174-362 are cited from the literature(Dong et al., 2013)

Table S6. The subgroup distribution of CDS haplotypes (gcHaps).

	Hap1	Hap2	Hap3	Hap4	Hap5	Hap6	Hap7	Hap8	Hap9	Hap10	Hap11
Aus	2	2	3	0	0	79	0	75	0	0	21
Bas	64	0	3	0	0	1	1	0	0	0	1
Adm	29	9	10	2	4	7	3	2	1	0	3
XI	53	483	457	170	160	38	113	30	50	39	8
GJ	806	11	9	0	1	0	1	0	0	0	0
total	954	505	482	172	165	125	118	107	51	39	33

Table S7. The subgroup distribution of 1-kb promoter haplotypes (gpHaps).

	Hap1	Hap2	Hap3	Hap4	Hap5	Hap6	Hap7	Hap8	Hap9	Hap10	Hap11
Aus	54	0	24	0	0	16	0	0	0	0	1
Bas	0	40	1	0	0	1	1	0	0	0	0
Adm	13	10	2	1	4	6	4	2	1	0	4
XI	332	4	139	25	126	99	1	56	43	39	0
GJ	8	324	1	116	1	3	101	0	0	0	26
total	407	378	167	142	131	125	107	58	44	39	31

Table S8. Likelihood ratio test statistic and parameters estimated from the M0 and mF Models in PAML.

Model	np	InL	Parameters	Models Compared	2△L	p-Value
M0	84	-60962.0886	ω0=0.0935	M0 vs. Mf	50.3174	<0.001
Mf	165	-60253.4181	**ω1=2.7397⋆**			

⋆ Branch of H2
